# Repurposing Multiple-Molecule Drugs for COVID-19-Associated Acute Respiratory Distress Syndrome and Non-Viral Acute Respiratory Distress Syndrome via a Systems Biology Approach and a DNN-DTI Model Based on Five Drug Design Specifications

**DOI:** 10.3390/ijms23073649

**Published:** 2022-03-26

**Authors:** Ching-Tse Ting, Bor-Sen Chen

**Affiliations:** Laboratory of Automatic Control, Signaling Processing and Systems Biology, Department of Electrical Engineering, National Tsing Hua University, Hsinchu 30013, Taiwan; rich870811@gmail.com

**Keywords:** COVID-19, SARS-CoV-2, HPI-GWGEN, host-pathogen RNA-Seq data, non-viral ARDS, biomarkers, etiologic mechanism, DTI model, deep neural network, systems biology

## Abstract

The coronavirus disease 2019 (COVID-19) epidemic is currently raging around the world at a rapid speed. Among COVID-19 patients, SARS-CoV-2-associated acute respiratory distress syndrome (ARDS) is the main contribution to the high ratio of morbidity and mortality. However, clinical manifestations between SARS-CoV-2-associated ARDS and non-SARS-CoV-2-associated ARDS are quite common, and their therapeutic treatments are limited because the intricated pathophysiology having been not fully understood. In this study, to investigate the pathogenic mechanism of SARS-CoV-2-associated ARDS and non-SARS-CoV-2-associated ARDS, first, we constructed a candidate host-pathogen interspecies genome-wide genetic and epigenetic network (HPI-GWGEN) via database mining. With the help of host-pathogen RNA sequencing (RNA-Seq) data, real HPI-GWGEN of COVID-19-associated ARDS and non-viral ARDS were obtained by system modeling, system identification, and Akaike information criterion (AIC) model order selection method to delete the false positives in candidate HPI-GWGEN. For the convenience of mitigation, the principal network projection (PNP) approach is utilized to extract core HPI-GWGEN, and then the corresponding core signaling pathways of COVID-19-associated ARDS and non-viral ARDS are annotated via their core HPI-GWGEN by KEGG pathways. In order to design multiple-molecule drugs of COVID-19-associated ARDS and non-viral ARDS, we identified essential biomarkers as drug targets of pathogenesis by comparing the core signal pathways between COVID-19-associated ARDS and non-viral ARDS. The deep neural network of the drug–target interaction (DNN-DTI) model could be trained by drug–target interaction databases in advance to predict candidate drugs for the identified biomarkers. We further narrowed down these predicted drug candidates to repurpose potential multiple-molecule drugs by the filters of drug design specifications, including regulation ability, sensitivity, excretion, toxicity, and drug-likeness. Taken together, we not only enlighten the etiologic mechanisms under COVID-19-associated ARDS and non-viral ARDS but also provide novel therapeutic options for COVID-19-associated ARDS and non-viral ARDS.

## 1. Introduction

The coronavirus disease 2019 (COVID-19) is a novel pandemic caused by the new coronavirus severe acute respiratory syndrome coronavirus 2 (SARS-CoV-2). Since mid-July 2021, there have been more than 183 million cases and 3.9 million deaths around the world due to the rapid spread of COVID-19 [1]. SARS-CoV-2-infected patients have demonstrated a wide spectrum of clinical manifestations. Although the majority (81%) of COVID-19 patients experienced mild symptoms (e.g., asymptomatic, flu-like symptoms, or mild pneumonia), 14% of cases experienced severe symptoms (e.g., dyspnea or hypoxemia), around 5% of COVID-19 patients were critically ill (e.g., multiple organ failure or septic shock), and about 20% of COVID-19 patients required hospitalization [2,3,4,5].

Acute respiratory distress syndrome (ARDS), the severe form of acute lung injury (ALI), is an acute respiratory failure syndrome resulting from noncardiogenic lung edema and hypoxemia [6]. Common causes of ARDS developments can be infective (viral or bacterial pneumonia) or non-infective (e.g., pancreatitis and trauma). ARDS is also a frequent complication in COVID-19. Among hospitalized COVID-19 patients, about 30~40% of patients develop ARDS, 26% require intensive care unit (ICU) facilities, and 16% receive intermittent mandatory ventilation (IMV). Furthermore, for the ICU COVID-19 patients, 75% have ARDS. The mortality rate of COVID-19-associated ARDS patients approximately ranges from 26% to 61.5% [7,8,9,10]. The high incidence and mortality ratio observed among COVID-19-associated ARDS cases indicate that there is an urgent need to develop relative pharmaceutical therapies. Comparisons of clinical characteristics and pathophysiology between COVID-19-associated ARDS and classical ARDS (not associated with SARS-CoV-2) are still under debate. Most of the recent evidence suggest that there is no significant difference regarding respiratory compliance, lung morphology, and myocardial injury [11]. Some studies have also indicated that COVID-19-associated ARDS has higher coagulation potential and thromboembolic complications risk [12,13]. However, their corresponding molecular pathogenetic mechanisms and the role of epigenetics and genetic factors between COVID-19-associated ARDS and classical ARDS (not associated with SARS-CoV-2) are not fully understood.

The microRNAs (miRNA) are short, non-protein-coding, and single-stranded RNA with 18–25 nucleotides in length. After binding to the 3′-untranslated region (3′UTR) or 5′-untranslated region (5′UTR) of mRNA transcripts, microRNAs can post-transcriptionally control gene expression either by mRNA degradation or directly inhibiting the translation process [14,15]. Given that miRNAs can control some biological activities in multi-levels such as cell proliferation, apoptosis, and even immune responses during virus infection, several studies have been dedicated to elucidating the complicated pathogenesis and epigenetic interplay between SARS-CoV-2 and humans. Several dysregulated miRNAs observed in differential gene analysis results have also been identified as biomarkers and proposed as therapeutic targets for COVID-19. In addition, the discovery of SARS-CoV-2 encoded miRNAs that can target human genes has also been investigated, although it is controversial because RNA viruses are mainly replicated in the cytoplasm and miRNA production may interfere with the replication of the viral genome. Several machine-learning-based bioinformatics tools and databases have been developed to predict virus-encoded miRNA and possible targets of human genes [16,17,18]. 

Long noncoding RNAs (lncRNAs) are another type of functional, non-protein-coding RNA longer than 200 nucleotides. By interacting with mRNA, DNA, or transcription factors, lncRNAs engage in versatile biological events such as modulating gene expression, epigenetic modification [19,20]. Increasing evidence has shown that lncRNAs play important roles during SARS-CoV-2 infection. For example, recent studies indicated that lncRNAs NEAT1 and MALAT1 are associated with immune responses in SARS-CoV-2 infected cells [21,22].

In traditional drug discovery, the average period of new drug development pipelines takes at least 12 years from the initial discovery to the marketplace [23]. Although the pharmaceutical industry invested 83 billion USD worldwide on research and development (R&D) expenditures in 2019 [24], the success rate of a drug candidate starting from clinical trial to marketing approval was approximately 10~20%, which has not changed for the past few decades [25]. On the contrary, drug repurposing (also known as drug repositioning), which aims to identify new therapeutic uses of approved or investigational drugs, is a feasible and advantageous strategy with a lower development risk and time cost. To this end, numerous approaches for drug repurposing have been developed, including experimental models, retrospective clinical analysis, virtual screening, signature-based methods, pathway mapping, etc. [26]. Additionally, combination therapies deployed with repurposed drugs have also been considered as therapeutic interventions for COVID-19. At present, thousands of repurposed clinical trials are being tested for COVID-19 [27,28,29]. Although most of them are monotherapy, the importance of accelerating the evaluation efficacy should not be neglected. 

**Figure 1:**
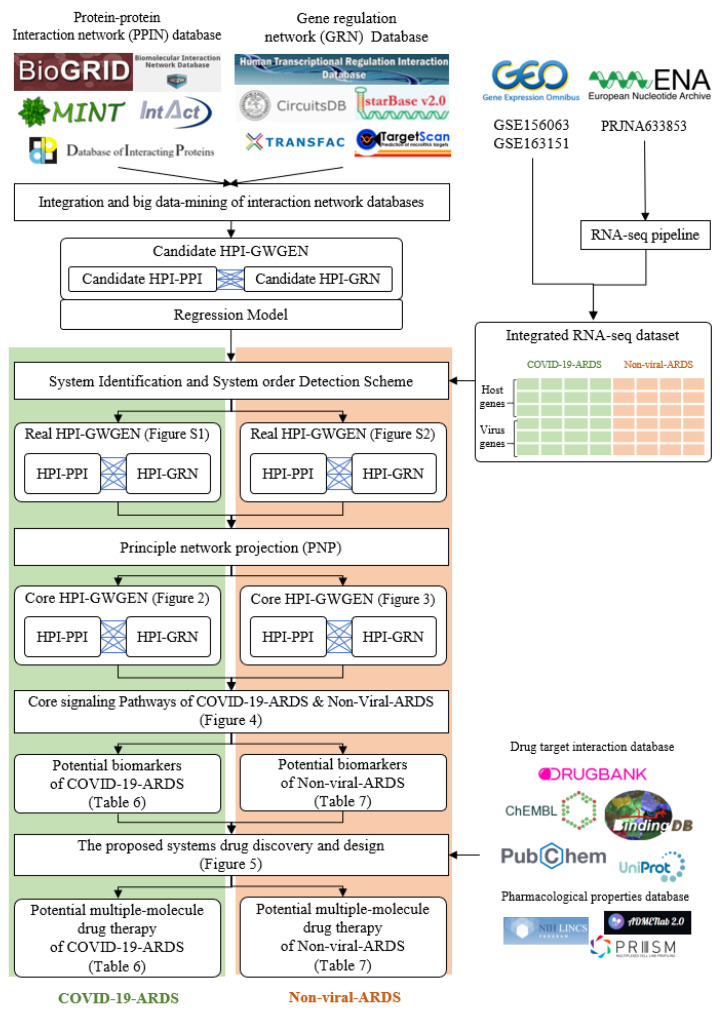
The flowchart for constructing candidate HPI-GWGEN, real HPI-GWGEN, core HPI-GWGEN, and core signaling pathways for biomarker identification for systems drug discovery and design of potential multiple-molecule drugs for therapeutic treatment of COVID-19-associated ARDS and non-viral ARDS.

In this study, we established a workflow, shown in Figure 1, which utilizes a systems biology approach to investigate pathogenetic mechanisms to identify essential biomarkers as drug targets, and selected potential compounds as multiple-molecule drugs for the therapy of COVID-19 by the training of a deep neural network as a drug–target interaction (DTI) model and through the filtering of drug specifications. First of all, a candidate host-pathogen interspecies genome-wide genetic and epigenetic interaction network (HPI-GWGEN) was constructed by big data mining from molecular interaction databases. Secondly, with the information collected from candidate HPI-GWGEN and host-pathogen RNA-Seq datasets of COVID-19-associated ARDS and non-viral ARDS, we built system models describing all possible interaction conditions for each gene, protein, and epigenetics to simultaneously identify the best model’s parameters to obtain real HPI-GWGENs by Akaike Information Criterion (AIC) system order detection method. Thirdly, by applying the principal network projection (PNP) method and based on the ranking projection value calculated for each gene protein and epigenetics, we extracted core HPI-GWGENs from real HPI-GWGENs. By the denotation of KEGG pathways, we could obtain the core signaling pathways from the corresponding core HPI-GWGEN. Meanwhile, by investigating the malfunctions in the core pathways and downstream cellular functions, the essential biomarkers of COVID-19-associated ARDS and non-viral ARDS could be identified as drug targets, respectively. Then, a deep neural network (DNN) is trained as the drug–target interaction (DTI) model by drug target interaction databases for these essential biomarkers (drug targets) to predict candidate drugs. Finally, based on drug design specifications including drug regulation ability, high sensitivity, adequate excretion, low toxicity, and drug-likeness as selection criteria, we narrowed down candidate drugs predicted by the DNN-DTI model and proposed the multiple-molecule drugs as the therapeutic recommendation for clinical trials of COVID-19-associated ARDS and non-viral ARDS, respectively.

## 2. Results

### 2.1. Overview of Core HPI-GWGEN Construction and Drug Discovery Design for COVID-19-Associated ARDS and Non-Viral ARDS by Systems Biology Approach

**Figure 2:**
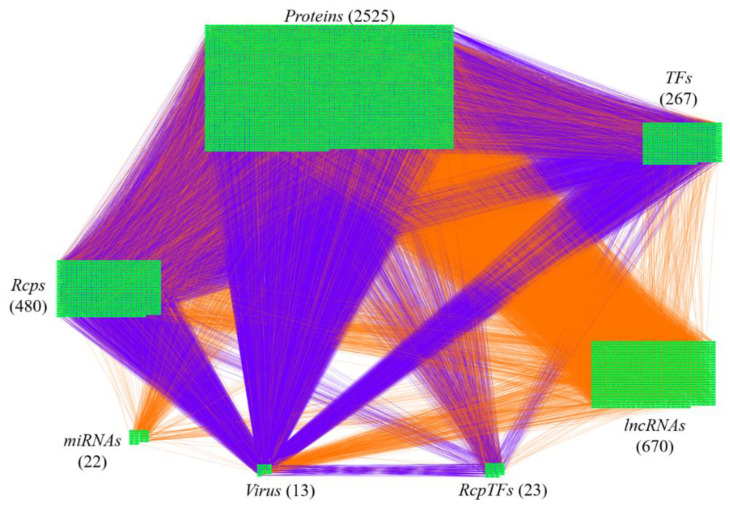
The core host-pathogen interspecies genome-wide genetic and epigenetic network (core HPI-GWGEN) of COVID-19-associated ARDS. Purple lines indicate the protein–protein interactions and orange lines denote the gene regulations. The node numbers of *proteins, Rcps, TFs, RcpTFs, miRNA, LncRNA, Virus* are 2525, 480, 23, 22, 670, and 13, respectively.

**Figure 3:**
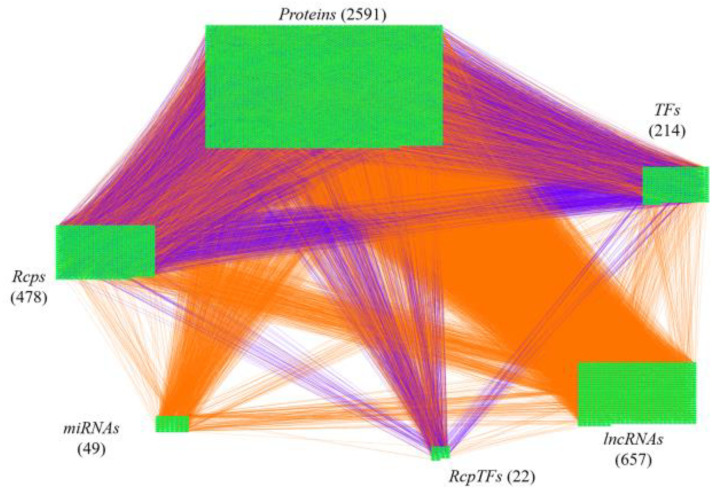
The core host-pathogen interspecies genome-wide genetic and epigenetic network (core HPI-GWGEN) of non-viral ARDS. Purple lines indicate the protein–protein interactions and orange lines denote the gene regulations. The nodes numbers of *proteins, Rcps, TFs, RcpTFs, miRNA, LncRNA* are 2591, 487, 214, 22, 49, and 657, respectively.

**Figure 4:**
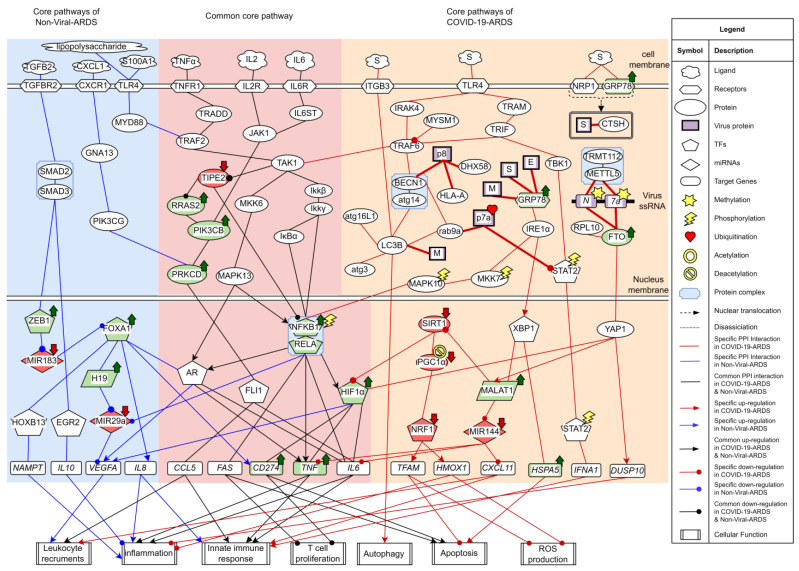
The common and specific core signaling pathways between COVID-19-associated ARDS and non-viral ARDS. This figure summarizes the genetic and epigenetic progression mechanism of COVID-19-associated ARDS and non-viral ARDS. The blue color background covers specific signaling pathways in non-viral ARDS. Overlapping core signaling pathways of COVID-19-associated ARDS and non-viral ARDS, namely common core signaling pathways, are covered in pink background. The skin color background covers specific signaling pathways in COVID-19-associated ARDS. The arrowheads in circle shapes indicate downregulation. The arrowheads in triangular shapes indicate upregulation. The solid lines indicate protein–protein interaction. The green nodes indicate high expression of protein/gene. The red nodes indicate low expression of protein/gene.

The research flowchart, as shown in Figure 1, is used to summarize how to construct candidate HPI-GWGEN, real HPI-GWGEN, core HPI-GWGEN, and core signaling pathways of COVID-19-associated ARDS and non-viral ARDS. Sample groups and statistics of the node of COVID-19-associated ARDS and non-viral ARDS are described in Table 1. Essentially, the candidate HPI-GWGEN we integrated by database mining is a data structure of binary matrix to represent if there is any interaction (edge) between two arbitrary genes/proteins (nodes), which can be encoded from either human or virus. According to the edge’s types, candidate HPI-GWGEN can be further subdivided into HPI-PPI (host-pathogen interspecies protein–protein interaction between two nodes) and HPI-GRN (host-pathogen interspecies gene regulation between two nodes). Since these databases we integrated only recorded the existence between two nodes, such information may depend on actual detection expression levels and be different from person to person. Thus, there are false positive interactions among the interactions within candidate HPI-GWGEN which needs to be trimmed off by the real host/pathogen RNA-Seq data. To deal with this issue, assisting with the integrated RNA-Seq datasets, as shown in Table 1, and candidate HPI-GWGEN, for each node, we simultaneously constructed all possible regression system models. Each model represents the potential interaction relationships of each node with other nodes and the fitting interaction parameters of HPI-GWGEN can be estimated by the constrained least-square parameter identification method by the real host/pathogen RNA-Seq data. Real HPI-GWGEN can be obtained by trimming off the false positive interactions out of the system order of each node identified by the Akaike information criterion (AIC). We used system matrix A of real HPI-GWGEN in Equation (25) to store these evaluated parameters of each node. Statistic information of candidate HPI-GWGEN, i.e., real HPI-GWGEN of COVID-19-associated ARDS and non-viral ARDS are shown in Table 2 and Table 3, respectively. Real HPI-GWGENs of COVID-19-associated ARDS and non-viral ARDS were also visualized by Cytoscape software (version 3.8.2) [30], as shown in Figures S1 and S2. One could find that the total nodes and edges in real HPI-GWGEN from both groups are significantly smaller than the candidate HPI-GWGEN, indicating that false positive interactions of each protein/gene were trimmed successfully. The real HPI-GWGENs of COVID-19-associated ARDS and non-viral ARDS are still very complex and not easy for further analysis. For the convenience of analysis, we further extracted core HPI-GWGENs of COVID-19-associated ARDS and non-viral ARDS to reduce network size via selecting significant nodes by applying the principal network projection (PNP) method in Equations (27)–(29). The core HPI-GWGENs based on 4000 significant nodes of COVID-19-associated ARDS and non-viral ARDS visualized by Cytoscape software (version 3.8.2) [30] are shown in Figure 2 and Figure 3, respectively. In the meantime, for the top 4000 nodes in core HPI-GWGENs of COVID-19-associated ARDS and non-viral ARDS, we also utilized DAVID Bioinformatics Resources (2021 update) [31] to obtain the enrichment analysis of Kyoto Encyclopedia of Genes and Genomes (KEGG) pathways annotation and correlative cellular functions, as shown in Tables S2 and S3, respectively. On the basis of referencing literature surveys and the KEGG signaling pathways annotation, we obtained core signaling pathways of COVID-19-associated ARDS and non-viral ARDS. Then, through investigating the common and specific core signaling pathways between COVID-19-associated ARDS and non-viral ARDS in Figure 4, we identified common specific biomarkers of infection pathogenesis as drug targets, which were TNF, NFκB, HIF1A, GRP78, FTO, and BECN1 (in Table 6) for COVID-19-associated ARDS and TNF, NFκB, HIF1A, and FOXA1 (in Table 7) for non-viral ARDS. 

Afterward, we trained a DTI model of DNN by drug–target interaction data in advance. By the use of the DNN-DTI model, we obtained a binary classifier, with a high probability to predict potential candidate drugs for these drug targets of COVID-19-associated ARDS and non-viral ARDS, through holding higher probability values of interactions with drug targets. Given these candidate drugs (in Table 4), we further narrowed them down by considering drug specifications (i.e., regulation ability, sensitivity, excretion, toxicity in Table 4, and drug-likeness in Table 5). Consequently, with these candidate drugs and their corresponding drug targets, we suggested two multiple-molecule drugs composed of nicorandil, isoliquiritigenin, eugenol, and omeprazole for COVID-19 and nicorandil, bortezomib, and olaparib for non-viral ARDS, as shown in Table 6 and Table 7, respectively. Detailed discussions of the above results are described in the following subsections. 

### 2.2. The Common Pathogenic Molecular Mechanism between COVID-19-Associated ARDS and Non-Viral ARDS

From the first common signaling pathway related to inflammation, as shown in Figure 4, after interacting with microenvironment factor TNFa, receptor TNFR1 can activate TAK1 by signaling through TRADD and TRAF2. Among all the transcription factors in the downstream pathways of TAK1, TF NFκB stood out to be the most pivotal component governing the inflammation. Initiated by the Ikkβ/Ikkγ, IκBα undergoes phosphorylation-induced degradation, resulting in the translocation of TF NFκB into the nucleus to induce its target genes *TNF, IL6,* and *FAS*. The target genes *TNF* and *IL6* both encode critical proinflammatory cytokines eliciting inflammation and innate immune responses [32,33]. Upregulated by TF NFκB, TF HIF1α can induce target genes *CD274, TNF,* and *IL6*. The target genes *CD274* and *FAS* both contribute to the inhibition of T lymphocyte proliferation and promote apoptosis [34,35,36]. Adequate amounts and differentiated lymphocytes are prerequisites for activating the adaptive immune response. Triggering apoptosis in activated lymphocytes is commonly used as a means of controlling the ongoing inflammation. Exhausted and low levels of lymphocytes often lead to a condition known as lymphopenia, which has been reported in ARDS [37]. Especially in the COVID-19-associated ARDS cases, the development of lymphopenia might derive from multiple mechanisms that work together and several hypotheses have been proposed. Observations from several basic studies have supported that dysregulated expression of proinflammatory cytokines including tumor necrosis factor (TNFα) and interleukin (IL-6) might lead to lymphocyte apoptosis [38,39,40,41,42]. Apart from that, high-level expressions of FAS (CD95) and CD274 could contribute to the exhaustion and depletion of T cells [43,44]. Furthermore, viruses might also directly infect lymphocytes expressing ACE2 [45]. Upregulated TF HIF1A is a critical indicator in response to cellular hypoxia conditions. The inflammation role of TF HIF1α has also been investigated in ARDS caused by different agents, suggesting that silencing HIF1 depends on NFκB and could be a possible strategy for preventing the aggravation of inflammation in ARDS [46,47,48,49].

Additionally, TAK1 can also stimulate the MAPK signaling pathway comprised of MKK6/MAPK13. Typically, androgen receptor (AR) belongs to the nuclear receptor family that has the dual role of functioning as transcription factors. Apart from being activated through steroids-mediated induction, transcription factor AR can also be phosphorylated by kinases involved in the signaling transduction pathway and provoke the expression of cytokine-related target genes *TNF* and *IL6*, such behavior has been commonly described in several cancer researches [50,51]. In this study, transcription factor AR links with MAPK13 (p38 delta) and contributes to inflammation.

Lack of negative regulator of immune response may also contribute to the hyperinflammation of cytokine. From the core common signaling pathways, as shown in Figure 4, we demonstrated that TNF alpha induced protein 8 like 2 (TIPE2), a negative regulator considered to modulate the NFKB and MAPK signaling pathways, can inhibit Ras signaling effector Ras2 to downregulate PI3KCB. One study indicated that PRKCD could be phosphorylated by PI3KCB, confirming this downstream interactor of PI3KCB [52]. PRKCD can further interact with transcription factor FLI1 to induce the target genes *CCL5* and *IL6* [53,54,55]. CCL5(RANTES), encoded by gene *CCL5*, is a chemokine contributing to leukocyte recruitment in innate immune responses [56]. It is noticed that there is a relatively lower expression of TIPE2, whereas relative higher expressions of its downregulated proteins were observed, signifying that the inhibitory effect of TIPE2 may be attenuated. Since there also exists an upstream interaction between TAK1 and TIPE2 in this study, it is reasonable to suppose that TIPE2 ubiquitination may contribute to the loss-of-control cytokine production [57].

Collectively, the common molecular mechanisms in COVID-19-associated ARDS and non-viral ARDS are leukocyte recruitments, inflammation, innate immune responses, apoptosis, and T cell inhibition. Based on the results of core signaling analyses and considering relative protein/gene expression levels as compared with normal nasopharyngeal tissues [58], we choose TNF, NFkB, and HIF1A as common biomarkers (drug targets) of infections pathogenesis in both COVID-19-associated ARDS and non-viral ARDS.

### 2.3. The Specific Pathogenic Molecular Mechanism of COVID-19-Associated ARDS

The early stage of the SARS-CoV-2 life cycle begins from the attachment of the host cellular receptor and the membrane fusion between virus and host cell. Accomplishments of both events are required for releasing viral RNA into the cytoplasm for the subsequent replication and translation. Although, currently, it has been effectively established that angiotensin-converting enzyme 2 (ACE2) is the main receptor for SARS-CoV-2 cell entry [59], there is no stop to identifying novel receptors that may potentiate the SARS-CoV-2 infectivity. 

Several cell receptors are identified to interact with the Spike protein of SARS-CoV-2 in Figure 4. Firstly, ITGB3, an integrin protein thought to contain an LC3-interacting region (LIR), can bind to LC3 and contribute to autophagy upon activation [60]. In agreement with the previous studies that the toll-like receptor (TLR) signaling pathway can be triggered by structural proteins of SARS-CoV-2 [61,62,63]. After recognizing the Spike protein of SARS-CoV-2, receptor TLR4 could transmit the signal to TRAF6 by recruitment of adaptor proteins either IRAK4 or TRAM/TRIF. TRAF6 could promote proinflammatory cytokines expression by activating downstream pathways of TAK1 and NFκB as aforementioned. TF STAT2, phosphorylated by TBK1, can promote innate immune response by activating its target gene *IFNA1* [64]. However, ORF7a of SARS-CoV-2 has been found to interact with STAT2 as well. A recent study showed that attenuation of this type-I interferon (IFN-I) signaling pathway may be attributed to the ubiquitination of ORF7a [65]. NRP-1, a receptor widely expressed in nasal and olfactory tissue, was intended to interact with the Spike protein of SARS-CoV-2, coinciding with the current studies [66,67]. On top of that, the Spike protein was also found to interact with cathepsin H (CTSH). Functional cleavage of the Spike protein of SARS-CoV-2 by endosomal protease cathepsin is a necessary process for membrane fusion. In contrast to thoroughly studied CTSL and CTSB, there are few studies in the literature that refer to the relation between CTSH and SARS-CoV-2 [68]. GRP78 (Bip), a chaperone originally resident in the endoplasmic reticulum (ER) lumen, can not only ensure protein proper folding but also be a major stress sensor maintaining the homeostasis of ER folding capacity by triggering unfolded protein responses (UPR). In Figure 4, upon interacting with structural proteins of SARS-CoV-2, GRP78 was identified to stimulate IRE1α and TF XBP1. TF XBP1 can promote transcription of GRP78 encoded by gene *HSPA5*. Under the stress caused by the accumulation of unfolded viral proteins, one measure to resolve the stress is to further promote chaperone production in the downstream signaling pathways of UPR. Emerging research has reported that high expression levels of GRP78 and apoptosis are observed in SARS-CoV-2 infected cells [69,70,71]. Overexpressed GRP78 has been observed to translocate to the cell membrane, further facilitating virus entry by interacting with S proteins of coronaviruses, including SARS-CoV-2 [72,73]. The positive feedback loop of GRP78 production established by virus infection may eventually lead to the sustained UPR and subsequent apoptosis. Moreover, IRE1α also contributes to inflammation by transmitting the signal through MKK7 and MAPK10.

The higher expression level of lncRNA metastasis-associated lung adenocarcinoma transcript 1 (MALAT1/NEAT2) has been considered to have a critical role in inflammation and cytokine production. Similar results were also observed in saliva and nasopharyngeal swabs of COVID-19 patients [74], however, details of the mechanisms of MALAT1 upregulation and the cytokine production mediated by MALAT1 in COVID-19 have not been well illustrated. Herein, we identified TF XBP1 as one of the upstream nodes of lncRNA MALAT1. A previous bioinformatic analysis has indicated that TF XBP1 binding site exists within the MALAT1 gene promoter region [75], suggesting that MALAT1 upregulation may be due to endoplasmic reticulum (ER) stress and unfolded protein response (UPR) induction [76]. MALAT1 has been confirmed to downregulate miRNA MIR144 [77], and miRNA MIR144 has been shown to suppress the expression of cytokines and chemokines, including TNFα, IL6, and *CXCL11* [78,79,80]. It can also suppress the TRAF6 level post-transcriptionally [81]. Notably, the lower expression of MIR144 is also observed, which is consistent with the differential expression analysis in the peripheral blood of COVID-19 patients [82]. It is possible that MALAT1 can promote cytokine production through MIR-144. By acting as a transcriptional coactivator, YAP1 can induce the expression of MALAT1 and also stabilize TF HIF1α [83,84]. Furthermore, YAP1 can interact with dual-specificity phosphatase 10 (DUSP10/MKP5) [85]. Dual-specificity phosphatases are well known to be negative regulators of YAP1 on p38 and MAPK pathways [86].

Additionally, YAP1 can serve as a node connecting inflammation and the N^6^-methyladenosine (m^6^A) modification system in COVID-19-associated ARDS. m^6^A is one of the host RNA modifications commonly used for epitranscriptomic control of cellular mRNAs. Recent studies have identified m^6^A in SARS-CoV-2 RNA, implying that the virus may utilize this machinery for its own benefit [87,88,89]. Several studies in the literature have reported the m^6^A inhibitory effect on SARS-CoV-2 replication. These modifications mediated by m^6^A “writer” protein METTL3 not only have an influence on the SARS-CoV-2 replication but also interfere with RIG-I binding, which is the key regulator of the cytosolic pattern recognition receptor (PRR) system [90]. However, conflicting results have also been observed, different from the well-documented results currently focused on the relationship between METTL3 and SARS-CoV-2. In Figure 4, the METTL5–TRMT112 complex was identified to interact with N and ORF7 genes in the core signaling pathway of COVID-19-associated ARDS. In addition, fat mass and obesity-associated protein (FTO), a m^6^A eraser protein, was also involved in the GRN interaction between N and ORF7 and found with higher expression levels as compared with normal nasopharyngeal tissues datasets. Interestingly, previous studies have shown that silencing the catalytic ability of demethylase FTO and ALKBH5 can drastically inhibit SARS-CoV-2 infection [89,91]. Furthermore, the depletion of fat mass and obesity-associated protein (FTO) can facilitate YAP1 mRNA degradation [92]. Overall, these results suggest that targeting m^6^A modification could be a potential therapeutic modality fighting against SARS-CoV-2.

Autophagy is an auto-degradative process conserved across eukaryotes and essential for maintaining intracellular homeostasis, which is characterized by forming autophagosome and later fusing with lysosome for degradation (known as autolysosome) [93]. Autophagosomes can also break down an invading pathogen by uptaking endosome after virus entry, thereby, contributing to part of the antiviral responses. It is known that double-membrane vesicles (DMVs) are a prerequisite for the replication of coronaviruses. Recently, it has been documented that SARS-CoV-2 infection induces the accumulation of autophagosomes [94]. Moreover, other reports have also observed that targeting autophagy led to the attenuation of SARS-CoV-2 replication [95,96]. Given that autophagosomes are double-membrane cellular compartments and the fact that nsp6 proteins of other coronaviruses family members colocalize with LC3 [97,98], it has been postulated that SARS-CoV-2 may also exploit the autophagy pathway for their life cycle [99]. In Figure 4, we showed that Beclin-1 (BECN1), which plays a critical role in the initiation of autophagy, can be activated by PPI interaction with TRAF6. The activation effect of TRAF6 on BECN1 has been confirmed in a previous study [100]. The relatively higher expression levels of BECN1 and its downstream protein LC3B can both be observed as compared with normal tissue datasets, which is consistent with several studies. Aside from that, SARS-CoV-2 can establish a more favorable intracellular environment by interfering with the autophagy process. For example, it has been reported that ORF8 was related to immune evasion by autophagy-mediated degradation of MHC-1 class family proteins, which was implicated in antigen processing and presentation [101]. As expected, BECN1-linked ORF8 was found to interact with HLA-A in this study. As a part of the RLRs (RIG-like receptors) pathogen recognition system, LGP2 (DHX58) has been thought to positively regulate MDA5/MAVS signaling. It has been documented that ectopic expression of SARS-CoV-2 ORF8 can suppress DHX58 basal level [102]. Herein, we speculate that the degradation of DHX58 mediated by ORF8 may partly contribute to this observation. Intriguingly, RAB9a, a GTPase mainly located in the late endosome and correlated with alternative autophagy, has been shown to interact with ORF7, implying that SARS-CoV-2 may interfere with autophagosome-lysosome fusion and reshape the morphology of *trans*-Golgi network (TGN) [103,104].

For the final core signaling pathway of COVID-19-associated ARDS, as shown in Figure 4. PGC1α, deacetylated by SIRT1, can activate TF NRF1 [105]. TF NRF1 can upregulate target gene *HMOX1* by interacting with its ARE element [106]. The heme oxygenase-1 (HO-1) encoded by *HMOX1* plays a protective role in oxidative tissue damage and its anti-inflammation effect in ARDS has been reported [107,108]. TF NRF1 can also target gene *TFAM*, which encodes TFAM that regulates the homeostasis of mitochondria. Similarly, previous studies have reported that downregulated SIRT1 and PGC1α were observed in COVID-19 patients [109,110,111]. We observed the lower expression level of SIRT1, which could be due to the inhibitory effect of MALAT1 [112]. Knockdown of target gene *TFAM* promotes reactive oxygen species (ROS) production and apoptosis [113]. Inhibition of TFAM in COVID-19 patients is not just limited to the transcriptional level. TFAM requires TOMM70 to translocate into mitochondria. A recent study observed that SARS-CoV-2 ORF9b interacted with TOMM70 [114]. The crystal structure of the complex of TOMM70 and SARS-CoV-2 ORF9b has also been resolved (PDB iD: 7KDT) [115], thereby, further inhibiting TFAM translocation into mitochondria. This may further dampen the result of absent TFAM.

In summary, according to the specific pathogenic pathways of COVID-19-associated ARDS, SARS-CoV-2 can hijack host factors to facilitate cell entry and modify the virus genome to promote virus protein transcription in the cytoplasm. Although innate immune systems such as interferon, sensor, or antigen-presenting protein system can be induced in response to virus invasion, their antiviral effects could be abrogated either by the virus proteins translated in the cytoplasm or autophagy-mediated degradation. ER signaling triggered by SARS-CoV-2 leads to abnormal cellular functions including leukocyte recruitments, inflammation, apoptosis, and ROS production, which are all critical driven factors for cytokine storm and the subsequent tissue damage in ARDS patients. Based on the results of core signaling analyses and considering relative protein/gene expression levels of COVID-19-associated ARDS as compared with normal nasopharyngeal human tissues [58], we selected GRP78, FTO, and BECN1 as essential biomarkers (drug targets) of specific etiologic mechanisms for COVID-19-associated ARDS.

### 2.4. The Specific Pathogenic Molecular Mechanism of Non-Viral ARDS

The core signaling pathways of non-viral ARDS are shown in Figure 4. Once stimulated by ligand S100A1 in the microenvironment, receptor TLR4 recruited adaptor protein MYD88 and promoted cytokine production by transmitting the signal through MAPK/NFKB axis, as previously described. S100A1 has been reported to contribute to hypoxia-induced inflammation in an earlier study. Although predominantly expressed in cardiomyocytes, S100A1 is also present in lung endothelium and its increased serum level has been documented in several pulmonary diseases [116,117,118,119]. The other ligand found to stimulate receptor TLR4 also includes lipopolysaccharide (LPS), which is the most studied molecule that constitutes the outer membrane of Gram-negative bacteria. 

Another specific core signaling pathway of non-viral ARDS is shown in Figure 4. As soon as the microenvironment molecule TGFB2 binds the receptor TGFBR2, it can activate the SMAD2/SMAD3 complex. SMAD2/SMAD3 can activate TF EGR2 to promote the inhibitory cytokine target gene *IL10* and also transmit the activation signal to TF ZEB1 [120,121,122]. MicroRNA MIR183 is the upstream node of TF FOXA1, however, TF ZEB1 can downregulate the transcription level of MIR183 [123]. In Figure 4, several downstream pathways of TF FOXA1 are shown to incorporate with the TGFβ signaling pathway. First, it has been validated that TF FOXA1 can induce the expression of target genes *CD274* and *IL8* by directly binding to their promoter region [124,125]. It is worth noting that the MIR183/FOXA1/CXCL8 pathway activated by HDAC2 has been investigated in a previous study [126]. In Figure 4, we show that this pathway is, instead, mediated by TF ZEB1 and incorporated with the downstream of the TGFβ signaling pathway. Secondly, in coordination with TF AR, TF FOXA1 can also induce the target genes *VEGFA, TNF,* and *IL6*. TF FOXA1 has been thought to be a pioneer protein to facilitate the target genes of TF AR by chromatin remodeling [127]. *VEGFA* encodes VEGF-A to increase vascular permeability and leukocyte recruitment [128,129]. It is also a direct target of TF HIF1α, and its expression can be inhibited by miRNA MIR29a [130,131]. Thirdly, TF FOXA1 has been found to bind to E1 enhancer of gene *H19* and correlates with lncRNA gene *H19* activation [132]; lncRNA H19 has been shown to suppress miRNA MIR29a [133]. Remarkably, in an ARDS mouse model induced by LPS, both expressions of FOXA1 and H19 were upregulated. Moreover, the knockdown of lncRNA H19 has been reported to attenuate inflammation and fibrosis by decreasing the mRNA level of TNF-α, IL-6, and VEGF [134]. Moreover, TF HOXB13 has also been identified to be upregulated by FOXA1. It has been demonstrated that HOXB13 can be upregulated by FOXA1, and the overexpression of HOXB13 has been shown to upregulate the expression of its target gene *NAMPT* [135,136]. The inflammation role of NAMPT has been investigated in an LPS-induced ALI mouse model [137].

In brief, the specific molecular mechanisms in non-viral ARDS are leukocyte recruitments, innate immune response, and inflammation. Based on the results of core signaling analyses and considering relative protein/gene expression levels of non-viral ARDS as compared with normal nasopharyngeal tissues [58], we additionally selected TF FOXA1 as an essential biomarker (drug target) of specific etiologic mechanisms for non-viral ARDS.

### 2.5. The Construction of Deep Neural Network as Drug–Target Interaction Model and Drug Specification Filters to Select Potential Small Compounds for Multiple-Molecule Therapies

**Figure 5:**
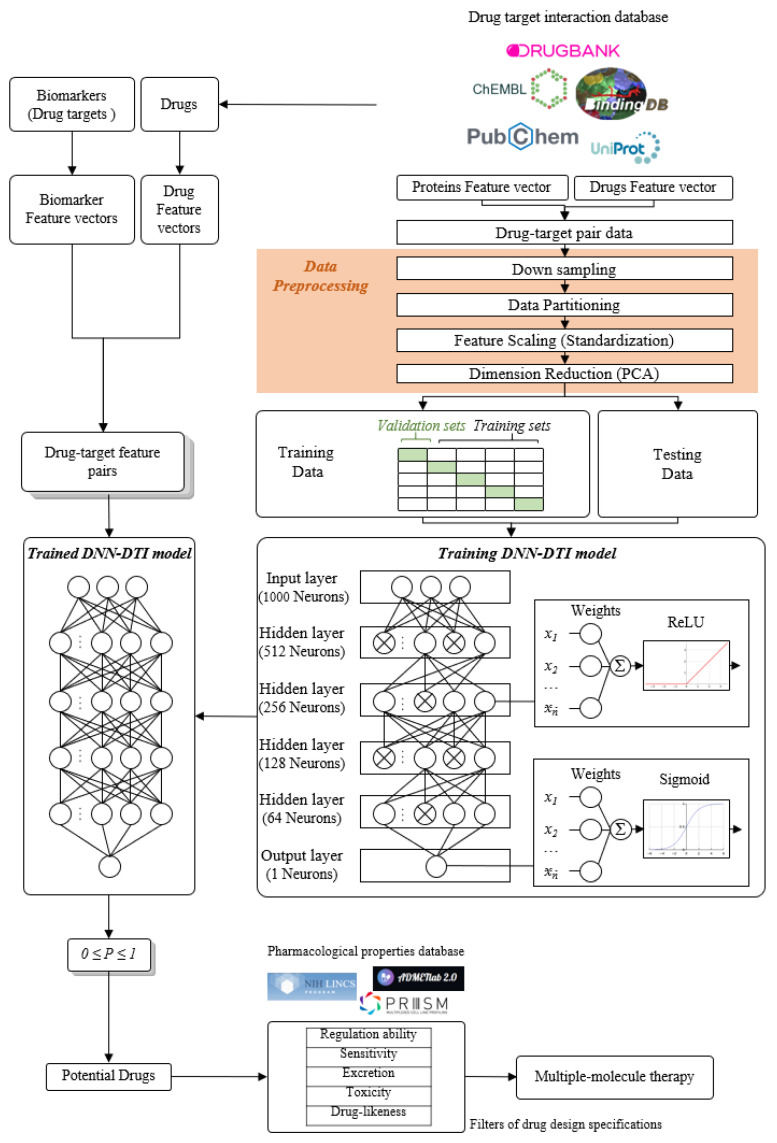
The flowchart for multiple-molecule drug design of COVID-19-associated ARDS and non-viral ARDS. In the right column, the drug–target interaction data are obtained from drug–target interaction databases to construct drug-target pair data. After data preprocessing, these data are divided into training data and testing data to train the DNN-DTI model for the trained DNN-DTI model in the left column. In the left column, the feature vectors of biomarkers and the feature vectors of drugs from drug–target interaction databases consist of drug-target feature pairs and are mounted into the trained DNN-DTI model to predict potential drugs for these biomarkers (drug targets). Then, these potential drugs are filtered by five drug design specifications to obtain candidate drugs as multiple-molecule drugs for COVID-19-associated ARDS and non-viral ARDS.

For the purpose of proposing potential multiple-molecule drugs to target identified biomarkers, we followed the design workflow, as shown in Figure 5, to train, in advance, a DNN-DTI model by drug–target interaction data. Drug–target interaction data for training a DNN-DTI model were collected from databases DrugBank [138], BindingDB [139], ChEMBL [139], UniProt [140], and PubChem [141]. There are two classes of drug-target pairs in our training datasets: 80,291 known drug–target interaction pairs (labeled with 1) and 100,024 unknown drug–target interaction pairs (labeled with 0). It is noted that the imbalanced datasets often cause a poor predictive performance, especially for the minority class. Therefore, the number of negative classes were randomly down sampled from 100,024 to 80,291. Moreover, each feature in the drug-target pairs is defined in different scales. Here, to make the DNN-DTI model learn well, we perform feature scaling by standardization. The data in high dimension space are often sparse such that model training is computationally intractable. Principal component analysis (PCA) [142] was adopted to reduce the number of features for each drug-target pair from 1359 to 1000. We split three-fourth of the datasets as training set and one-fourth as testing set. The training set was further subdivided five-fold with four-fifth for training and one-fifth for validation during model training. 

The architecture of the DNN-DTI model is composed of one input layer followed by four hidden layers and one output layer, as shown in Figure 5. Corresponding neuron numbers of inputs, hidden and output layer are 1000, 521, 256, 128, 64, 1, respectively. For each neuron of the hidden layer, ReLU was set as the activation function, while the sigmoid function was used for the output layer. We set the Adam learning algorithm [143] as an optimizer (learning rate 0.001, epoch 100, and batch size 100) and used binary cross-entropy as the loss function. To counter the overfitting, the early stopping strategy was employed to monitor the validation error at each epoch and stop model training once the error started to increase. Moreover, we set the dropout as 0.5 for each hidden layer. To avoid the bias caused by the particular combination of the dataset, we evaluated the DNN-DTI model performance by five-fold cross-validation. The learning curves of accuracy and loss are, respectively, shown in Figures S3 and S4. The average scores of validation loss, validation accuracy, testing loss, and testing accuracy were also calculated, when the training process of the DNN-DTI model automatically stopped at epoch 52, as presented in Table S4. Moreover, the receiver operating characteristic (ROC) curve of the DNN-DTI model with the area under the curve of ROC (AUC-ROC) score 0.982 is also provided in Figure S5.

### 2.6. Discovery of Multiple-Molecule Drug Therapy of COVID-19-Associated ARDS and Non-Viral ARDS

The candidate drugs of drug targets predicted by the DNN-DTI model were further filtered by the following five drug design specifications. For drug regulation ability, we downloaded Phase I L1000 level 5 datasets (GSE92742) from the Broad Institute Library of integrated Cellular Signatures (LINCS) [144,145]. This dataset includes the moderated Z-scores (MODZS) from differential gene analysis of 12*,*328 genes for 19*,*811 perturbagens (small molecule) treatments across 76 human cell lines corresponding with 45*,*956 expression signatures. The negative value of small molecules implies that abnormal gene overexpression in the cell line we choose can be downregulated under drug treatment, and vice versa. For each identified biomarker, the top five candidate drugs with suitable regulation ability, as mentioned above, are presented in Table 4. 

Afterward, we checked drug sensitivity. The corresponding dataset was obtained from DepMap Primary PRISM Repurposing datasets [146], consisting of chemical-perturbation viability screens for 4518 compounds across 578 human cell lines. We preferred to choose the compounds with sensitivity values around zeros, which meant that the cell line was not sensitive to the chemical perturbation. In addition, clearance (CL, mL/min/kg), toxicity (LC_50_, mol/kg), and drug-likeness were considered and evaluated using the web tool ADMETlab 2.0 [147]. Higher clearance (CL) indicates the drugs could be excreted easily and have fewer adverse effects on normal metabolism in the human body. Moreover, we preferred the drugs with higher LC_50_, implying the drug possessed a lower acute toxicity toward the body. Meanwhile, we also considered several drug-likeness rules commonly used in R&D to narrow down candidate drugs, based on a qualitative concept to determine whether compounds were similar to known drugs by evaluating their structural and physicochemical properties, including Lipinski rule [148], Pfizer rule [149], GSK rule [150]*,* and Golden Triangle [151] from ADMETlab 2.0. Definitions of these drug-likeness rules and their corresponding principle of choosing candidate drugs are listed in Table 5. Eventually, we proposed two multiple-molecule drugs for COVID-19-associated ARDS and non-viral ARDS, as shown in Table 6 and Table 7.

## 3. Discussion

### 3.1. Multiple-Molecule Drugs for COVID-19-Associated ARDS and Non-Viral ARDS

We investigated the core HPI-GWGENs of COVID-19-associated ARDS and non-viral ARDS with KEGG annotations, and described the common and distinctive core signaling pathways in detail (shown in Figure 4) in terms of the abnormal cellular functions and the interspecies cross-talk pathways that SARS-CoV-2 interfered with in the infectious process. With the application of a supervised-learning-based DNN-DTI model, we could predict interactions between candidate drugs and the identified drug targets. Meanwhile, considering drug design specifications including regulation ability, sensitivity, toxicity, and drug-likeness, we suggested multiple-molecule drugs for COVID-19-associated ARDS and non-viral ARDS, as shown, respectively, in Table 6 and Table 7. Among them, nicorandil (Ikorel^®^) is nicotinamide commonly used for the management of ischemia and angina pectoris due to its multi-pharmacological mechanism [152]. A recent in vivo study revealed that nicorandil could relieve oxidative stress, apoptosis, and inflammation in LPS-induced acute lung injury (ALI) mice by modulating the MAPK and NFkB pathways [153]. Nicorandil can suppress the release of TNFα from the immune cell line [154]. Another study also observed reduced HIF1A levels in palmary fibrosis rats after nicorandil treatment [155]. Isoliquiritigenin is a phytochemical flavonoid compound derived from licorice. Emerging evidence has suggested that isoliquiritigenin could suppress inflammasome and apoptosis by attenuating the NFkB pathway in a mouse model [156,157]. Isoliquiritigenin also demonstrated its efficacy to inhibit cell proliferation and migration of breast cancer by promoting HIF1A proteasome degradation [158,159]. Moreover, it has been reported that isoliquiritigenin can directly target GRP78 [160]. This observation was further supported by one molecular study, which indicated that isoliquiritigenin could fit into the ATPase domain of GRP78 [161]. Eugenol has been shown to reduce the TNFα expression in human macrophages induced by lipopolysaccharide (LPS) [162,163]. It has been documented that eugenol administration inhibited the accumulation of autophagosomes [164]. Omeprazole (Losec^®^, Prilosec^®^, Zegerid^®^, and others), a proton pump inhibitor, is commonly used to reduce gastrointestinal (GI) ulcers induced by nonsteroidal anti-inflammatory drugs (NSAIDs). It also exerts anti-inflammatory properties, as reported in a previous research [165]. Administration with omeprazole has been shown to decrease FTO level and, in turn, enhance the transcription level of the mechanistic target of rapamycin complex 1(mTORC1), which is a protein complex that regulates autophagy induction [166]. Bortezomib (Velcade), an anticancer drug commonly used as the standard treatment of multiple myeloma (MM), has been demonstrated to suppress NFκb activation owing to its ability of proteasome inhibition [167]. It can inhibit the FOXA1 stability by elevating the O-GlcNAc modification in the host cell [168]. Olaparib, a poly ADP ribose polymerase (PARP) inhibitor, has been used in the first-line treatment of patients with advanced ovarian cancer. Targeting PARPs could interrupt the interaction between TF FOXA1 and TF AR [169]. Overall, we proposed two multiple-molecule drugs: (1) nicorandil, isoliquiritigenin, eugenol, omeprazole for COVID-19-associated ARDS and (2) nicorandil, bortezomib, and olaparib for non-viral ARDS.

Currently, conventional medications for the treatment of ARDS such as statins and corticosteroids have been recommended for ARDS patients infected by SARS-CoV-2 [170,171]. However, several cohort studies and observational data suggest that the efficacy and safety of these drugs are controversial [172,173,174]. Questions regarding optimal dosage, treatment initiation, the time point of administration in the disease stage of COVID-19-associated ARDS patients have not reached a consensus and should be further discussed in clinical studies.

As compared with de novo drug design, drug repurposing aided with a systems biology approach seems to be a more promising proposition. It is worth noting that the multiple-molecule compounds that were selected for drug targets in this study are mainly U.S. Food and Drug Administration (FDA) approved drugs. On the one hand, repurposing FDA-approved drugs can greatly reduce the bottleneck during the development of traditional drugs, particularly, for emergency use to keep up with the pace of emerging outbreaks. On the other hand, by selecting multiple drugs with synergistic effects, the effective dosage of individual drugs can be reduced to prevent the possibility of toxicity. Although further clinical studies need to be validated, it is anticipated that COVID-19-associated ARDS and non-viral ARDS patients could benefit from multiple-molecule drug therapy.

### 3.2. The Limitations and Advantages to the Proposed Systems Medicine Design Procedure for COVID-19-Associated ARDS and Non-Viral ARDS

To the best of our knowledge, this is the first systematic study to discuss pathogenetic differences of host-pathogen interactome between COVID-19-associated ARDS and non-viral ARDS from the systems biology perspective by leveraging both human and virus transcriptome data. The development of new treatments relies on an improved understanding of the underlying pathogenic mechanism of COVID-19-associated ARDS and non-viral ARDS. With more and more relative studies being conducted, significant efforts have been made to find more accurate treatments to attenuate virus replication and pathogen-derived complications. Nonetheless, most SARS-CoV-2-related datasets known from the GEO database have limited sample sizes and only focus on the host transcriptomic responses. To date, effective technology that measures the genome-scale protein expression profile of both humans and SARS-CoV-2 has not been established. Increasing evidence has shown that cellular protein abundance can be estimated by their corresponding mRNA, implying that RNA-Seq data can substitute protein expressions and provide sufficient information for solving the constrained least-squares problem in system identification method [175,176,177,178]. Thanks to the availability of RNA-Seq data of both humans and SARS-CoV-2, we can integrate these two-side datasets into a systems drug discovery design procedure to identify essential biomarkers, and then search for the plausible drug combination for the treatment of COVID-19-associated ARDS and non-viral ARDS patients. The systematic workflow in this study can also be applied to investigate other infectious diseases from the viewpoint of systems biology. Along with the expandability of public access data, it is also feasible to integrate additional databases via the proposed workflow, revealing a more comprehensive genetic and epigenetic network.

There are some drawbacks of the two RNA-Seq datasets we integrated in this study. Recently, outbreaks of SARS-CoV-2 variants have been raging around the globe at an increasing speed. However, due to the original study design of the RNA-Seq datasets, we only aligned RNA-Seq raw datasets on SARS-CoV-2 reference sequence (Ref-Seq) genome (NC_045512.2) to obtain virus gene count data. Hence, a discussion about the relationship between ARDS and SARS-CoV-2 variants is beyond the scope of this study. Furthermore, it is noted that ORF1ab transcripts are polyprotein precursors, which will be further cleaved by viral proteinases to produce 16 non-structural proteins (NSPs). However, only the ORF1ab transcripts level can be estimated from RNA-Seq data. Therefore, we only consider ORF1ab polyprotein rather than 16 NSPs to be one of *the “Virus”* protein names in HPI-PPI. How to estimate the proportions of each expression level of NSPs by ORF1ab transcripts remains to be a question.

## 4. Materials and Methods

### 4.1. Preprocessing of Host-Pathogen RNA-Seq Datasets and Construction of Candidate HPI-GWGEN by RNA-Seq Pipeline and Big-Data Mining

In this study, both human and virus gene count data of multiple studies from the GEO database were integrated before systematic model construction. After evaluating the overall design and availability of data, two GEO datasets (accession nos. GSE156063 [179] and GSE163151 [180]) were retrieved according to the following screening criteria in this study: (1) Samples of datasets can be classified as COVID-19-associated ARDS and non-viral ARDS; (2) at least one study provides RNA-Seq raw data, RNA-Seq raw data of GSE156063 (PRJNA633853) can be accessed and batch downloaded from the European Nucleotide Archive (ENA) database; (3) nasopharyngeal (NP) swab sample specimens; (4) identical sequencing platform (GPL24676).

Technical details of the RNA-Seq process pipeline to obtain GSE156063 virus gene count data are as follows: (1) Trim adapters and filter low-quality reads by fastp tool [181], (2) align reads on the SARS-CoV-2 reference sequence (Ref-Seq) genome (NC_045512.2) by HISAT2 tool [182], (3) assemble alignments and gene count calculation by StringTie tool [183]. Tools are all included in the Subio Software (version 1.24.5849) and default options were used to perform the automatic process. 

Since two selected datasets were tabular format (gene names in rows and samples in columns), we classified gene names in each dataset into seven classes according to their functions (Table 1). Data integration is mainly considered in two aspects: (1) gene names of integrated data in each class are the union of two selected datasets of the corresponding sets, (2) integrated data samples are the union of two selected datasets of the corresponding groups. For each original dataset we selected, if gene count data in the sample group were not provided for integrated gene names, the missing value were estimated from the distribution across the corresponding sample group in the other dataset. Data distributions were fitted by kernel density estimation (KDE) with normal kernel and the optimal bandwidth that minimized the mean integrated squared error (MISE). Finally, the integrated gene count data were normalized to transcripts per million units (TPM) for downstream analysis.

Among the candidate HPI-GWGEN, candidate human protein–protein interactions (HPI-PPIs) were obtained from the Database of Interacting Proteins (DIP) [184], the Biomolecular Interaction Network Database (BIND) [185], the Biological General Repository for Interaction Datasets (BIOGRID) [186], IntAct [187], and the Molecular INTeraction Database (MINT) [188]. Candidate human gene-regulation networks (HPI-GRNs) were obtained from the Human Transcriptional Regulation Interactions database (HTRIdb) [189], the Integrated Transcription Factor Platform database (ITFP) [190], the Target Scan Human database [191], StarBase2.0 [192], CircuitDB [193], and the TRANScription FACtor database (TRANSFAC) [194]. Considering that the information of host-pathogen PPI and GRN current databases may not be fully discovered and sufficient for the constructions of candidate host-virus PPIs, candidate host-virus GRNs, candidate virus PPIs, and candidate virus GRNs, we assumed each virus protein/gene could interact with each other in default to prevent false negative interactions at first, and then trimmed false positive interactions by host-pathogen RNA-Seq data via the proposed system model identification and AIC system order detection.

### 4.2. Systematic Model Construction for the Candidate HPI-GWGEN of COVID-19-Associated ARDS and Non-Viral ARDS Patients

The *i*th host protein PPI interaction model can be described by the following equation:

(1) $$\begin{array}{c} p_{i}^{H}\left[n\right]=\sum_{\kappa =1,\kappa \neq i}^{K_{i}}K_{i\kappa }^{H}p_{i}^{H}\left[n\right]p_{\kappa }^{H}\left[n\right]+\sum_{v=1}^{V_{i}}V_{iv}^{H}p_{i}^{H}\left[n\right]p_{v}^{p}\left[n\right]+\beta_{i,PPI}^{H}+\varepsilon_{i,PPI}^{H}\left[n\right]\\ for\;i=1\sim I,n=1\sim N \end{array}$$

where $p_{i}^{H}\left[n\right],p_{\kappa }^{H}\left[n\right],\;p_{v}^{P}\left[n\right]$ indicate the expression level of the *i*th host protein, the *κ*th host protein, and the νth pathogen protein in the nth sample, respectively; $K_{i\kappa }^{H},\;V_{iv}^{H}$ indicate the interaction ability between the *i*th host protein and κth host protein and between the *i*th host protein and the νth pathogen protein, respectively; $\beta_{i,PPI}^{H}$ indicates the basal level of the *i*th host protein in the nth sample; $\varepsilon_{i,PPI}^{H}\left[n\right]$ indicates stochastic noise of the *i*th host protein in the nth sample;$K_{i},V_{i}$ indicate the total number of host proteins and pathogen proteins interacting with the *i*th host protein, respectively; I indicates the total number of the *i*th host protein in candidate PPI; N denotes sample number in candidate PPI, either in COVID-19-ARDS or Non-Viral-ARDS group.

The qth pathogen protein PPI interaction model can be described by the following equation:

(2) $$\begin{array}{c} p_{q}^{P}\left[n\right]=\sum_{\kappa =1}^{K_{q}}K_{q\kappa }^{P}p_{q}^{P}\left[n\right]p_{\kappa }^{H}\left[n\right]+\sum_{v=1,\nu \neq q}^{V_{q}}V_{qv}^{P}p_{q}^{P}\left[n\right]p_{v}^{P}\left[n\right]+\beta_{q,PPI}^{P}+\varepsilon_{q,PPI}^{P}\left[n\right]\\ for\;q=1\sim Q,n=1\sim N \end{array}$$

where $p_{q}^{p}\left[n\right],\;p_{\kappa }^{H}\left[n\right],\;p_{v}^{p}\left[n\right]$ indicate the expression level of the qth pathogen protein, the *κ*th host protein, and the νth pathogen protein in the nth sample, respectively; $K_{q\kappa }^{P},\;V_{qv}^{P}$ indicate the interaction ability between the qth pathogen protein and κth host protein and between the qth pathogen protein and the νth pathogen protein, respectively; $\beta_{q,PPI}^{H}$ indicates the basal level of the qth pathogen protein in the nth sample; $\varepsilon_{q,PPI}^{H}\left[n\right]$ indicates the stochastic noise of the qth host protein in the nth sample; $K_{q}\;,V_{q}$ indicate the total number of host proteins and pathogen proteins interacting with the qth pathogen protein, respectively; *Q* indicates the total number of the qth pathogen protein in the candidate PPI; *N* denotes the sample number in the candidate PPI, either in COVID-19-ARDS or Non-Viral-ARDS group.

For the host HPI-GRNs in HPI-GWGEN, the *j*th host gene GRN interaction model can be described by the following equation:

(3) $$\begin{array}{c} g_{j}^{H}\left[n\right]=\sum_{\tau =1,\tau \neq j}^{T_{j}}T_{j\tau }^{H}t_{\tau }^{H}\left[n\right]-\sum_{\mu =1}^{M_{j}}M_{j\mu }^{H}g_{j}^{H}\left[n\right]m_{\mu }^{H}\left[n\right]+\sum_{\lambda =1}^{L_{j}}L_{j\lambda }^{H}l_{\lambda }^{H}\left[n\right]+\beta_{j,GRN}^{H}+\varepsilon_{j,GRN}^{H}\left[n\right]\\ -M_{j\mu }^{H}\leq 0\;\;\;\;\;for\;j=1\sim J,n=1\sim N \end{array}$$

where $g_{j}^{H}\left[n\right],t_{\tau }^{H}\left[n\right],l_{\lambda }^{H}\left[n\right],m_{\mu }^{H}\left[n\right]$ indicate the expression level of the *j*th host gene, the τth host TF, the *λ*th host lncRNA gene, and the μth host miRNA gene in the nth sample, respectively; $T_{j\tau }^{H},\;L_{u\lambda }^{H},\;M_{j\mu }^{H}$ indicate the regulation ability of the τth host TF, the λth host lncRNA gene and the μth host miRNA gene on the *j*th host gene, respectively; $\beta_{j,GRN}^{H}$ indicates the basal level of the *j*th host gene in the nth sample; $\varepsilon_{j,GRN}^{H}\left[n\right]$ indicates stochastic noise of the *j*th host gene in the nth sample; $T_{j},L_{j},M_{j}$ indicate the total number of host TF, host lncRNA gene and host miRNA gene interacting with the *j*th host gene, respectively; *J* indicates the total number of the *j*th host gene in candidate GRN; *N* denotes sample number in candidate GRN, either in COVID-19-ARDS or Non-viral-ARDS group. Similar concept of systems modeling on miRNAs and lncRNAs could be found in Supplementary Materials. 

Numerous studies have suggested that SARS-CoV-2 proteins affect host gene expression mainly by inhibiting upstream human TFs. To regulate host genes by direct association (binding) with DNA, pathogen proteins should possess a nucleic acid binding domain and be able to enter the nucleus. Although one study showed that SARS-CoV-2 RNA-binding proteins (RBPs) could be detected in nuclear and colocalized with SC35 [195], the study was conducted by viral transfection which may be different from viral infection. More experimental evidence should be provided to support that SARS-CoV-2 RBPs can bind to the host gene. Therefore, we excluded the regulation between the host gene and pathogen proteins in Equation (3).

The uth pathogen gene GRN interaction model can be described by the following equation:

(4) $$\begin{array}{c} g_{u}^{P}\left[n\right]=\sum_{\tau =1}^{T_{u}}T_{u\tau }^{P}t_{\tau }^{H}\left[n\right]+\sum_{f=1,f\neq u}^{F_{u}}F_{uf}^{P}t_{f}^{P}\left[n\right]-\sum_{\mu =1}^{M_{u}}M_{\mu u}^{P}g_{u}^{P}\left[n\right]m_{\mu }^{H}\left[n\right]+\sum_{\lambda =1}^{L_{u}}L_{u\lambda }^{P}l_{\lambda }^{H}\left[n\right]+\beta_{u,GRN}^{P}+\varepsilon_{u,GRN}^{P}\left[n\right]\\ -M_{ju}^{P}\leq 0\;\;\;\;\;for\;u=1\sim U,n=1\sim N \end{array}$$

where $g_{u}^{P}\left[n\right],t_{f}^{P}\left[n\right],t_{\tau }^{H}\left[n\right],l_{\lambda }^{H}\left[n\right],m_{\mu }^{H}\left[n\right]$ indicate the expression level of the uth pathogen gene, the fth pathogen TF, the τth host TF, the lth host lncRNA gene, and the μth host miRNA gene in the nth sample, respectively; $F_{uf}^{P}\;,\;T_{u\tau }^{P},\;L_{u\lambda }^{P},\;M_{u\mu }^{P}$ indicate the regulation ability of the fth pathogen TF,τth host TF, the λth host lncRNA gene and the μth host miRNA gene on the uth pathogen gene, respectively; $\beta_{u,GRN}^{P}$ indicates the basal level of the uth pathogen gene in the nth sample; $\varepsilon_{u,GRN}^{H}\left[n\right]$ indicates the stochastic noise of the uth pathogen gene in the nth sample; $F_{u},T_{u},L_{u},M_{u}$ indicate the total number of pathogen TF, host TF, host lncRNA gene, and host miRNA gene interacting with the uth pathogen gene, respectively; *U* indicates the total number of the uth host gene in candidate GRN; *N* denotes sample number in candidate GRN, in either the COVID-19-associated ARDS or non-viral ARDS group.

### 4.3. Parameter Estimation of Real HPI-GWGENs of COVID-19-Associated ARDS and Non-Viral ARDS by System Identification, System Order Detection Methods, and RNA-Seq Data

To identify interactive parameters of HPI-PPI and HPI-GRN in the real HPI-GWGEN for COVID-19-associated ARDS and non-viral ARDS, respectively, we applied the system identification method to estimate parameters of HPI-GWGEN by host-pathogen RNA-Seq data after stochastic model construction. Equations (1)–(4) can be expressed as the following regression form, respectively:

(5) $$\begin{array}{c} p_{i}^{H}\left[n\right]=\left[\begin{array}{ccccccc} p_{i}^{H}\left[n\right]p_{1}^{H}\left[n\right] & \cdots & p_{i}^{H}\left[n\right]p_{K_{i}}^{H}\left[n\right] & p_{i}^{H}\left[n\right]p_{1}^{p}\left[n\right] & \cdots & p_{i}^{H}\left[n\right]p_{V_{i}}^{p}\left[n\right] & 1 \end{array}\right]\times \left[\begin{array}{c} K_{i1}^{H}\\ \vdots \\ K_{iK_{i}}^{H}\\ V_{i1}^{H}\\ \vdots \\ V_{iV_{i}}^{H}\\ \beta_{i,PPI}^{H} \end{array}\right]+\varepsilon_{i,PPI}^{H}\left[n\right]\\ =\varphi_{i}^{HP}\left[n\right]\theta_{i}^{HP}+\varepsilon_{i,PPI}^{H}\left[n\right],for\;i=1\sim I,n=1\sim N \end{array}$$

(6) $$\begin{array}{c} p_{q}^{P}\left[n\right]=\left[\begin{array}{ccccccc} p_{q}^{P}\left[n\right]p_{1}^{H}\left[n\right] & \cdots & p_{q}^{P}\left[n\right]p_{K_{q}}^{H}\left[n\right] & p_{q}^{P}\left[n\right]p_{1}^{p}\left[n\right] & \cdots & p_{i}^{p}\left[n\right]p_{V_{i}}^{p}\left[n\right] & 1 \end{array}\right]\times \left[\begin{array}{c} K_{q1}^{P}\\ \vdots \\ K_{qK_{q}}^{p}\\ V_{q1}^{p}\\ \vdots \\ V_{qVq}^{p}\\ \beta_{q,PPI}^{p} \end{array}\right]+\varepsilon_{q,PPI}^{P}\left[n\right]\\ =\varphi_{q}^{PP}\left[n\right]\theta_{q}^{PP}+\varepsilon_{q,PPI}^{P}\left[n\right],for\;q=1\sim Q,n=1\sim N \end{array}$$

(7) $$\begin{array}{c} g_{j}^{H}\left[n\right]=\left[\begin{array}{cccccccccc} t_{1}^{H}\left[n\right] & \cdots & t_{T_{j}}^{H}\left[n\right] & g_{J}^{H}\left[n\right]m_{1}^{H}\left[n\right] & \cdots & g_{j}^{H}\left[n\right]m_{M_{j}}^{H}\left[n\right] & l_{1}^{H}\left[n\right] & \cdots & l_{L_{j}}^{H}\left[n\right] & 1 \end{array}\right]\times \left[\begin{array}{c} T_{j1}^{H}\\ \vdots \\ T_{jT_{J}}^{H}\\ -M_{j1}^{H}\\ \vdots \\ -M_{jM_{j}}^{H}\\ L_{j1}^{H}\\ \vdots \\ L_{jL_{j}}^{H}\\ \beta_{j,GRN}^{H} \end{array}\right]+\varepsilon_{j,GRN}^{H}\left[n\right]\\ =\varphi_{j}^{HG}\left[n\right]\theta_{j}^{HG}+\varepsilon_{j,GRN}^{H}\left[n\right],for\;j=1\sim J,n=1\sim N \end{array}$$

(8) $$\begin{array}{c} g_{u}^{P}\left[n\right]=\left[\begin{array}{ccccccccccccc} t_{1}^{H}\left[n\right] & L & t_{T_{u}}^{H}\left[n\right] & t_{1}^{P}\left[n\right] & L & t_{F_{u}}^{P}\left[n\right] & g_{u}^{P}\left[n\right]m_{1}^{H}\left[n\right] & L & g_{u}^{P}\left[n\right]m_{M_{u}}^{H}\left[n\right] & l_{1}^{H}\left[n\right] & L & l_{L_{u}}^{H}\left[n\right] & 1 \end{array}\right]\times \left[\begin{array}{c} T_{u1}^{P}\\ \vdots \\ T_{uT_{u}}^{P}\\ F_{u1}^{P}\\ \vdots \\ F_{uF_{u}}^{P}\\ -M_{u1}^{P}\\ \vdots \\ -M_{uM_{u}}^{H}\\ L_{u1}^{P}\\ \vdots \\ L_{\mu L_{\mu }}^{P}\\ \beta_{u,GRN}^{P} \end{array}\right]+\varepsilon_{u,GRN}^{P}\left[n\right]\\ =\varphi_{u}^{PG}\left[n\right]\theta_{u}^{PG}+\varepsilon_{u,GRN}^{P}\left[n\right],for\;u=1\sim U,n=1\sim N \end{array}$$

where the superscripts *H, P, HP, PP, HG,* and *PG* denote abbreviations of the host, pathogen, host protein, pathogen protein, host gene, and pathogen gene, respectively; $\varphi_{i}^{HP}[n],\;\varphi_{q}^{PP}[n],\;\varphi_{j}^{HG}[n],\;\varphi_{u}^{PG}\left[n\right]$ denote the regression vectors which can be obtained from the corresponding expression data we integrated; $\theta_{i}^{HP},\theta_{q}^{PP},\theta_{j}^{HG},\theta_{u}^{PG}$ are corresponding unknown parameter vectors of the *i*th host protein, the qth pathogen protein, the *j*th host gene, and the uth pathogen gene, respectively.

Equations (5)–(8) can be further augmented for N samples as follows:

(9) $$\left[\begin{array}{c} p_{i}^{H}\left[1\right]\\ p_{i}^{H}\left[2\right]\\ \vdots \\ p_{i}^{H}\left[N\right] \end{array}\right]=\left[\begin{array}{c} \varphi_{i}^{HP}\left[1\right]\\ \varphi_{i}^{HP}\left[2\right]\\ \vdots \\ \varphi_{i}^{HP}\left[N\right] \end{array}\right]\theta_{i}^{HP}+\left[\begin{array}{c} \varepsilon_{i,PPI}^{H}\left[1\right]\\ \varepsilon_{i,PPI}^{H}\left[2\right]\\ \vdots \\ \varepsilon_{i,PPI}^{H}\left[N\right] \end{array}\right]\;\;\;for\;i=1\sim I$$

(10) $$\left[\begin{array}{c} p_{q}^{P}\left[1\right]\\ p_{q}^{P}\left[2\right]\\ \vdots \\ p_{q}^{P}\left[N\right] \end{array}\right]=\left[\begin{array}{c} \varphi_{q}^{PP}\left[1\right]\\ \varphi_{q}^{PP}\left[2\right]\\ \vdots \\ \varphi_{q}^{PP}\left[N\right] \end{array}\right]\theta_{q}^{PP}+\left[\begin{array}{c} \varepsilon_{q,PPI}^{P}\left[1\right]\\ \varepsilon_{q,PPI}^{P}\left[2\right]\\ \vdots \\ \varepsilon_{q,PPI}^{P}\left[N\right] \end{array}\right]\;\;\;for\;q=1\sim Q\;$$

(11) $$\left[\begin{array}{c} g_{j}^{H}\left[1\right]\\ g_{j}^{H}\left[2\right]\\ \vdots \\ g_{j}^{H}\left[N\right] \end{array}\right]=\left[\begin{array}{c} \varphi_{j}^{HG}\left[1\right]\\ \varphi_{j}^{HG}\left[2\right]\\ \vdots \\ \varphi_{j}^{HG}\left[N\right] \end{array}\right]\theta_{j}^{HG}+\left[\begin{array}{c} \varepsilon_{j,GRN}^{H}\left[1\right]\\ \varepsilon_{j,GRN}^{H}\left[2\right]\\ \vdots \\ \varepsilon_{j,GRN}^{H}\left[N\right] \end{array}\right]\;\;\;for\;j=1\sim J\;$$

(12) $$\left[\begin{array}{c} g_{u}^{P}\left[1\right]\\ g_{u}^{P}\left[2\right]\\ \vdots \\ g_{u}^{P}\left[N\right] \end{array}\right]=\left[\begin{array}{c} \varphi_{u}^{PG}\left[1\right]\\ \varphi_{u}^{PG}\left[2\right]\\ \vdots \\ \varphi_{u}^{PG}\left[N\right] \end{array}\right]\theta_{u}^{PG}+\left[\begin{array}{c} \varepsilon_{u,GRN}^{P}\left[1\right]\\ \varepsilon_{u,GRN}^{P}\left[2\right]\\ \vdots \\ \varepsilon_{u,GRN}^{P}\left[N\right] \end{array}\right]\;\;\;for\;u=1\sim U$$

Equations (9)–(12) above can be simply represented as follows:

(13) $$P_{i}^{H}=\Phi_{i}^{HP}\theta_{i}^{HP}+\Omega_{i}^{HP},for\;i=1\sim I$$

(14) $$P_{q}^{P}=\Phi_{q}^{PP}\theta_{q}^{PP}+\Omega_{q}^{PP},for\;q=1\sim Q\;$$

(15) $$G_{j}^{H}=\Phi_{j}^{HG}\theta_{j}^{HG}+\Omega_{j}^{HG},for\;j=1\sim J\;$$

(16) $$G_{u}^{P}=\Phi_{u}^{PG}\theta_{u}^{PG}+\Omega_{u}^{PG},for\;u=1\sim U\;$$

For each parameter vector $\theta_{i}^{HP},\theta_{q}^{PP},\theta_{j}^{HG},\theta_{u}^{PG}$ in Equations (13)–(16), we can individually estimate by solving the constrained least-square problem as follows:

(17) $${\tilde{\theta }}_{i}^{HP}=\underset{\theta_{i}^{HP}}{argmin}\frac{1}{2}{\left\|\Phi_{i}^{HP}\theta_{i}^{HP}-P_{i}^{H}\right\|}_{2}^{2}$$

(18) $${\tilde{\theta }}_{q}^{PP}=\underset{\theta_{q}^{PP}}{argmin}\frac{1}{2}{\left\|\Phi_{q}^{PP}\theta_{q}^{PP}-P_{q}^{P}\right\|}_{2}^{2}$$

(19) $$\begin{array}{l} {\tilde{\theta }}_{j}^{HG}=\underset{\theta_{j}^{HG}}{argmin}\frac{1}{2}{\left\|\Phi_{j}^{HG}\theta_{j}^{HG}-G_{j}^{H}\right\|}_{2}^{2},subject\;to\;A_{j}^{HG}{\tilde{\theta }}_{j}^{HG}\leq B_{j}^{HG}\\ where\;A_{j}^{HG}=\left[\begin{array}{cccc} O_{M_{j}\times T_{j}}^{} & I_{M_{j}\times M_{j}}^{} & O_{M_{j}\times L_{j}}^{} & O_{M_{j}\times 1}^{} \end{array}\right],\;B_{j}^{HG}=\left[O_{M_{j}\times 1}^{}\right] \end{array}$$

(20) $$\begin{array}{l} {\tilde{\theta }}_{u}^{PG}=\underset{\theta_{u}^{PG}}{argmin}\frac{1}{2}{\left\|\Phi_{u}^{PG}\theta_{u}^{PG}-G_{u}^{P}\right\|}_{2}^{2},subject\;to\;A_{u}^{PG}{\tilde{\theta }}_{u}^{PG}\leq B_{u}^{PG}\\ where\;A_{u}^{PG}=\left[\begin{array}{ccccc} O_{M_{u}\times F_{u}}^{} & O_{M_{u}\times T_{u}}^{} & I_{M_{u}\times M_{u}}^{} & O_{M_{u}\times L_{u}}^{} & O_{M_{u}\times 1}^{} \end{array}\right],\;B_{u}^{PG}=\left[O_{M_{u}\times 1}^{}\right] \end{array}$$

where O and I denote zero matrix and identity matrix, respectively.

It is noted that in the parameter fitting process for the regression model of each protein/gene, what candidate HPI-GWGEN provided is all the possible binding molecules, and therefore our model needs further parameter trimming process. However, such a model parameter identification process in Equations (17)–(20) will often result in overfitting conditions with a finite sample of the dataset at hand. Therefore, the AIC detection method was employed to detect the system order (i.e., the number of interactions of each protein with other proteins or the number of regulator TFs on each gene by the fact that system order can minimize the corresponding AIC) [196,197]. For each model in HPI-GWGEN, the AIC values of the *i*th host protein in Equation (1), the qth pathogen protein in Equation (2), the *j*th host gene in Equation (3), and the uth pathogen gene in Equation (4) are defined as follows:

(21) $$AIC_{i}^{HP}({\tilde{\theta }}_{i}^{HP},\Phi_{i}^{HP},P_{i}^{H})=log(\frac{{\left\|\Phi_{i}^{HP}{\tilde{\theta }}_{i}^{HP}-P_{i}^{H}\right\|}_{2}^{2}}{N})+\frac{2dim({\tilde{\theta }}_{i}^{HP})}{N},\;for\;i=1\sim I$$

(22) $$AIC_{q}^{PP}({\tilde{\theta }}_{q}^{PP},\Phi_{q}^{PP},P_{q}^{P})=log(\frac{{\left\|\Phi_{q}^{PP}{\tilde{\theta }}_{q}^{PP}-P_{q}^{P}\right\|}_{2}^{2}}{N})+\frac{2dim({\tilde{\theta }}_{q}^{PP})}{N},\;for\;q=1\sim Q$$

(23) $$AIC_{j}^{HG}({\tilde{\theta }}_{j}^{HG},\Phi_{j}^{HG},G_{j}^{H})=log(\frac{{\left\|\Phi_{j}^{HG}{\tilde{\theta }}_{j}^{HG}-G_{j}^{H}\right\|}_{2}^{2}}{N})+\frac{2dim({\tilde{\theta }}_{j}^{HG})}{N},\;for\;j=1\sim J$$

(24) $$AIC_{u}^{PG}({\tilde{\theta }}_{u}^{PG},\Phi_{u}^{PG},G_{u}^{P})=log(\frac{{\left\|\Phi_{u}^{PG}{\tilde{\theta }}_{u}^{PG}-G_{u}^{P}\right\|}_{2}^{2}}{N})+\frac{2dim({\tilde{\theta }}_{u}^{PG})}{N},\;for\;u=1\sim U$$

where $dim({\tilde{\theta }}_{i}^{HP}),dim({\tilde{\theta }}_{q}^{PP}),dim({\tilde{\theta }}_{j}^{HG}),\;anddim({\tilde{\theta }}_{u}^{PG})$ denote the parameter vector dimension of each model, respectively. In general, increasing parameter number (system order) will result in good model fit, such that log residual error in the first term of AIC will decrease and the second term of AIC will increase, and vice versa. Therefore, there should be exact parameter numbers as system order to achieve the minimum AIC among all possible binding combinations for each protein/gene. Considering practical computational efficiency for implementation, for each protein/gene, forward and backward stepwise algorithms were both adopted to find the minimum AIC in Equations (21)–(24), with the corresponding parameter numbers to achieve the minimum AIC in Equations (17)–(20) with the help of *lsqlin* function in 2021 MATLAB optimization toolbox. Therefore, we trimmed the insignificant parameters in candidate HPI-GWGEN out of system order detected by AIC to obtain real HPI-GWGEN of COVID-19-associated ARDS and non-viral ARDS. Likewise, system identification and system order selection method were applied to the miRNAs regulatory model and lncRNA regulatory model, which can be found in Supplementary Materials.

### 4.4. Extracting Core HPI-GWGEN from Real HPI-GWGEN by Using the PNP Approach

After trimming the false positives of candidate HPI-GWGEN to obtain the real HPI-GWGENs of COVID-19-associated ARDS and non-viral ARDS by the above systems biology method, it is still not easy to investigate the infections of COVID-19-associated ARDS and non-viral ARDS because their real HPI-GWGENs are still very complex. Therefore, the principal network projection (PNP) method was employed to extract their core HPI-GWGENs from the corresponding real HPI-GWGENs.

Before applying the PNP method to extract the core network from the real HPI-GWGEN, it was necessary to integrate the interactive and regulatory parameters we previously estimated into the system matrix. The system network matrix A of real HPI-GWGEN can be described as follow:

(25) $$\begin{array}{l} A=\left[\begin{array}{cccc} A_{HP,HP}^{} & A_{HP,PP}^{} & 0 & 0\\ A_{PP,HP}^{} & A_{PP,PP}^{} & 0 & 0\\ A_{HG,HP}^{} & 0 & A_{HG,HM}^{} & A_{HG,HL}^{}\\ A_{HM,HP}^{} & 0 & A_{HM,HM}^{} & A_{HM,HL}^{}\\ A_{HL,HP}^{} & 0 & A_{HL,HM}^{} & A_{HL,HL}^{}\\ A_{PG,HP}^{} & A_{PG,PP}^{} & A_{PG,HM}^{} & A_{PG,HL}^{} \end{array}\right]\\ =\left[\begin{array}{cccccccccccc} {\tilde{K}}_{11}^{H} & \cdots & {\tilde{K}}_{1I}^{H} & {\tilde{V}}_{11}^{H} & \cdots & {\tilde{V}}_{1Q}^{H} & 0 & \cdots & 0 & 0 & \cdots & 0\\ \vdots & {\tilde{K}}_{i\kappa }^{H} & \vdots & \vdots & {\tilde{V}}_{iv}^{H} & \vdots & \vdots & 0 & \vdots & \vdots & 0 & \vdots \\ {\tilde{K}}_{I1}^{H} & \cdots & {\tilde{K}}_{II}^{H} & {\tilde{V}}_{IQ}^{H} & \cdots & {\tilde{V}}_{IQ}^{H} & 0 & \cdots & 0 & 0 & \cdots & 0\\ {\tilde{K}}_{11}^{P} & \cdots & {\tilde{K}}_{1I}^{P} & {\tilde{V}}_{11}^{P} & \cdots & {\tilde{V}}_{1Q}^{P} & 0 & \cdots & 0 & 0 & \cdots & 0\\ \vdots & {\tilde{K}}_{q\kappa }^{P} & \vdots & \vdots & {\tilde{V}}_{qv}^{P} & \vdots & \vdots & 0 & \vdots & \vdots & 0 & \vdots \\ {\tilde{K}}_{Q1}^{P} & \cdots & {\tilde{K}}_{QI}^{P} & {\tilde{V}}_{Q1}^{P} & \cdots & {\tilde{V}}_{QQ}^{P} & 0 & \cdots & 0 & 0 & \cdots & 0\\ {\tilde{T}}_{11}^{H} & \cdots & {\tilde{T}}_{1I}^{H} & 0 & \cdots & 0 & -{\tilde{M}}_{11}^{H} & \cdots & -{\tilde{M}}_{1M}^{H} & {\tilde{L}}_{11}^{H} & \cdots & {\tilde{L}}_{1L}^{H}\\ \vdots & {\tilde{T}}_{j\tau }^{H} & \vdots & \vdots & 0 & \vdots & \vdots & -{\tilde{M}}_{j\mu }^{H} & \vdots & \vdots & {\tilde{L}}_{j\lambda }^{H} & \vdots \\ {\tilde{T}}_{J1}^{H} & \cdots & {\tilde{T}}_{IJ}^{H} & 0 & \cdots & 0 & -{\tilde{M}}_{J1}^{H} & \cdots & -{\tilde{M}}_{JM}^{H} & {\tilde{L}}_{J1}^{H} & \cdots & {\tilde{L}}_{JL}^{H}\\ {\tilde{T}}_{11}^{H} & \cdots & {\tilde{T}}_{1I}^{H} & 0 & \cdots & 0 & -{\tilde{M}}_{11}^{H} & \cdots & -{\tilde{M}}_{1M}^{H} & {\tilde{L}}_{11}^{H} & \cdots & {\tilde{L}}_{1L}^{H}\\ \vdots & {\tilde{T}}_{l\tau }^{H} & \vdots & \vdots & 0 & \vdots & \vdots & -{\tilde{M}}_{l\mu }^{H} & \vdots & \vdots & {\tilde{L}}_{l\lambda }^{H} & \vdots \\ {\tilde{T}}_{L1}^{H} & \cdots & {\tilde{T}}_{LI}^{H} & 0 & \cdots & 0 & -{\tilde{M}}_{L1}^{H} & \cdots & -{\tilde{M}}_{LM}^{H} & {\tilde{L}}_{L1}^{H} & \cdots & {\tilde{L}}_{LL}^{H}\\ {\tilde{T}}_{11}^{H} & \cdots & {\tilde{T}}_{1I}^{H} & 0 & \cdots & 0 & -{\tilde{M}}_{11}^{H} & \cdots & -{\tilde{M}}_{1M}^{H} & {\tilde{L}}_{11}^{H} & \cdots & {\tilde{L}}_{1L}^{H}\\ \vdots & {\tilde{T}}_{\mu \tau }^{H} & \vdots & \vdots & 0 & \vdots & \vdots & -{\tilde{M}}_{\mu x}^{H} & \vdots & \vdots & {\tilde{L}}_{\mu \lambda }^{H} & \vdots \\ {\tilde{T}}_{M1}^{H} & \cdots & {\tilde{T}}_{MI}^{H} & 0 & \cdots & 0 & -{\tilde{M}}_{M1}^{H} & \cdots & -{\tilde{M}}_{MM}^{H} & {\tilde{L}}_{M1}^{H} & \cdots & {\tilde{L}}_{ML}^{H}\\ {\tilde{T}}_{11}^{P} & \cdots & {\tilde{T}}_{1I}^{P} & {\tilde{F}}_{11}^{P} & \cdots & {\tilde{F}}_{1Q}^{P} & -{\tilde{M}}_{11}^{P} & \cdots & -{\tilde{M}}_{1M}^{P} & {\tilde{L}}_{11}^{P} & \cdots & {\tilde{L}}_{1L}^{P}\\ \vdots & {\tilde{T}}_{u\tau }^{P} & \vdots & \vdots & {\tilde{F}}_{uf}^{P} & \vdots & \vdots & -{\tilde{M}}_{\mu u}^{P} & \vdots & \vdots & {\tilde{L}}_{u\lambda }^{P} & \vdots \\ {\tilde{T}}_{U1}^{P} & \cdots & {\tilde{T}}_{UI}^{P} & {\tilde{F}}_{Q1}^{P} & \cdots & {\tilde{F}}_{UQ}^{P} & -{\tilde{M}}_{U1}^{P} & \cdots & -{\tilde{M}}_{UM}^{P} & {\tilde{L}}_{U1}^{P} & \cdots & {\tilde{L}}_{UL}^{P} \end{array}\right]\in {\mathbb{R}}^{\left(I+Q+J+L+M+U)\times (I+Q+M+L\right)} \end{array}$$

where *H, P, HP, PP, HG, HL, HM,* and *PG* denote the abbreviations of the host, pathogen, host protein, pathogen protein, host gene, host lncRNA gene, host miRNA gene, and pathogen gene, respectively; ${\tilde{K}}_{i\kappa }^{H},{\tilde{V}}_{iv}^{H}$ and ${\tilde{K}}_{q\kappa }^{P},{\tilde{V}}_{qv}^{P}$ in Equation (25) can be obtained by solving $\theta_{i}^{HP}$ in (17), $\theta_{q}^{PP}$in (18), and AIC parameter selection criteria in Equations (21) and (22), respectively; $\left\{{\tilde{T}}_{j\tau }^{H},-{\tilde{M}}_{j\mu }^{H},{\tilde{L}}_{j\lambda }^{H}\right\},\left\{{\tilde{T}}_{l\tau }^{H},-{\tilde{M}}_{l\mu }^{H},{\tilde{L}}_{l\lambda }^{H}\right\},\left\{{\tilde{T}}_{\mu \tau }^{H},-{\tilde{M}}_{\mu x}^{H},{\tilde{L}}_{\mu \lambda }^{H}\right\},\left\{{\tilde{T}}_{u\tau }^{P},{\tilde{F}}_{uf}^{P},-{\tilde{M}}_{\mu u}^{P},{\tilde{L}}_{u\lambda }^{P}\right\}$ in Equation (25) can be obtained by solving $\theta_{j}^{HG}$ in (19), $\theta_{u}^{PG}$ in (20), and AIC parameter selection criteria in Equations (23)–(24), respectively.

The principal network projection (PNP) method is an application of principal component analysis (PCA) to extract core elements in the system matrix *A* in Equation (25). System matrix *A* of real HPI-GWGEN can be represented by the singular value decomposition (SVD) as follows [198,199,200]:

(26) $$A=USV^{T}$$

where $U\in {\mathbb{R}}^{\left(I+Q+J+L+M+U\right)\times \left(I+Q+M+L\right)},V\in {\mathbb{R}}^{\left(I+Q+M+L\right)\times \left(I+Q+M+L\right)}$ and $S=diag(\sigma_{1},\ldots ,\sigma_{s},\ldots ,\sigma_{I+Q+M+L})\in {\mathbb{R}}^{\left(I+Q+M+L\right)\times \left(I+Q+M+L\right)}$ is a diagonal matrix composed of I+Q+M+L singular values of the system matrix *A* in nonincreasing order (i.e., $\sigma_{1}\geq \ldots \geq \sigma_{s}\geq \ldots \geq \sigma_{I+Q+M+L}$). We also introduced expression fraction $E_{w}$ to normalize each singular value:

(27) $$E_{w}=\frac{\sigma_{{}_{w}}^{2}}{\sum_{w=1}^{I+Q+M+L}\sigma_{{}_{w}}^{2}}$$

By selecting the minimum *X* such that $\sum_{w=1}^{X}E_{{}_{w}}^{}\geq 0.85$ from the energy perspective, we chose the top *X* singular values and corresponding *X* principal singular vectors composed of 85% energy of real HPI-GWGEN as the principal structure of HPI-GWGEN. After that, we introduced the projection value of each node (i.e., each real vector of A) in the real HPI-GWGEN to the top *X* singular vectors, sequentially, as follows:

(28) $$Proj_{R}\left(A_{row,i},V^{T*}\right)={\left[\sum_{k=1}^{X}{\left(A_{row,i}v_{k}\right)}^{2}\right]}^{\frac{1}{2}},for\;i=1,\ldots ,I+Q+J+L+M+U$$

where $A_{row,i}$ denote the *i*th row vector of system matrix *A*; $V^{*}andU^{*}$ are vector spaces spanned by the *X* principal singular vectors $\left\{v_{1},\ldots ,v_{X}\right\},\left\{u_{1},\ldots ,u_{X}\right\}$, respectively. The larger projection value in Equation (29) implies that the *i*th corresponding node is more significant in the HPI-GWGEN. Conversely, as projection value approaches zero, it implies that the *i*th corresponding node is not significant.

Next, the top 4000 nodes including human TFs, genes, miRNAs, lncRNAs, and pathogen proteins ranked with higher projection values in Equation (29) were selected to construct core HPI-GWGEN for COVID-19-associated ARDS and non-viral ARDS, respectively. We also uploaded these nodes to the DAVID website [31] to obtain KEGG pathway annotation for core signaling pathways of COVID-19-associated ARDS and non-viral ARDS, as shown in Tables S2 and S3. Eventually, we scrutinized the molecular pathogenic mechanism of COVID-19-associated ARDS and non-viral ARDS from their common and specific core signaling pathways in Figure 4 and selected the potential biomarkers in the table for drug discovery design.

### 4.5. Data Preprocess for the Deep Neuron Network-Based Drug–Target Interaction (DTI) Model in Multiple-Molecule Drug Design

After choosing significant biomarkers as potential drug targets for COVID-19-associated ARDS and non-viral ARDS, a systematic medicine design strategy was proposed to identify potential multiple-molecule drugs, as shown in Figure 5. First, the drug–target interaction data were integrated by mining databases from DrugBank [138], BindingDB [139], ChEMBL [201], UniProt [140], and PubChem [141]. Molecular descriptors are mathematical representations used to describe the physicochemical and structural interpretation of molecules. Since molecular descriptor can transform complicated molecule characteristics into a numerical value, the molecular descriptor is widely used for convenient and quantitative analysis in drug discovery such as molecular docking and quantitative structure–activity relationship (QSAR) studies. In view of this, we employed the functions of the PyBioMed [202] package to transform features of each drug and target into descriptor under Python 2.7 environment, respectively. The drug features we considered included 2D, 3D structural fingerprints, atomic constitution, topology, charges, etc. For target features, molecular descriptor calculated amino acid composition and sequence order, dipeptide and tripeptide composition, etc. For more details about descriptor transformation, please refer to PyBioMed documents [202].

Afterward, both descriptors of drugs and targets were concatenated into vector as DNN model datasets and can be described by the following form [203]:

(29) $$v_{Drug\text{-}Target}=\left[\begin{array}{cc} D_{} & T_{} \end{array}\right]=\left[\begin{array}{cccccccc} d_{1} & d_{2} & \cdots & d_{i} & t_{1} & t_{2} & \cdots & t_{j} \end{array}\right]\;\;\;for\;i=1\sim I,j=1\sim J$$

where $v_{Drug\text{-}Target}$ represents the feature vector for each drug-target pair; ***D*** represents the descriptor of the drug and *T* indicates the descriptor of corresponding target; *d_i_* denotes the *i*th drug feature and t_j_ denotes the *j*th target feature; *I* is the total feature number of the drug; *J* is the total feature number of the biomarker (drug targets).

### 4.6. Parameters Tuning Process and Prediction Quality Measurement of DNN-Based Drug Target Interaction Model

Basically, the training process of the deep neuron network in each iteration involved forward propagation and backpropagation steps. For the forward propagation step, each input data are fed in the network to output their corresponding probability value by sequential calculation from the input layer to the output layer. In the architecture of a deep neuron network, neuron calculations in each layer can be generalized by the following function:

(30) $${\tilde{h}}_{n}=\delta \left(w^{T}x_{n}+b\right)wherew=\left[\begin{array}{c} w_{1}\\ w_{2}\\ \vdots \\ w_{e} \end{array}\right],b=\left[\begin{array}{c} b_{1}\\ b_{2}\\ \vdots \\ b_{e} \end{array}\right]$$

where ${\tilde{h}}_{n}$ and $x_{n}$ represent the nth output and input vectors corresponding to the nth drug-target feature vector, respectively; δ(.) denotes activation function (ReLU function in hidden layer and Sigmoid function in output layer); *w* is weight vector and *b* is bias vector. 

With the output probability value calculated, the loss value can be obtained and the parameter set can be updated by computing the gradient of the loss function with respect to each weight during the backpropagation step [204]. Since drug–target interaction is a binary classification problem, we chose the cross-entropy function as the cost function to calculate the loss:

(31) $$L\left(y,\tilde{y}\right)=-\frac{1}{N}\sum_{n=1}^{N}\left\{y_{n}\log\left({\tilde{y}}_{n}\right)+\left(1-y_{n}\right)\log\left(1-{\tilde{y}}_{n}\right)\right\}=\frac{1}{N}\sum_{n=1}^{N}C^{n}\left(y,\tilde{y}\right)$$

where $y_{n}$ is the class label (1 for positive and 0 for negative); ${\tilde{y}}_{n}$ is the nth predicted probability value; $C^{n}\left(y,\tilde{y}\right)$ is the loss of the nth sample. In practice, a commonly used algorithm in the backpropagation step to find the gradient of the loss function and update the parameter set is the gradient descent method. The definition of the parameter set, and the update formulation are given as follows:

(32) $$\theta^{*}=\underset{\theta }{argmin}L\left(\theta \right)\;,\;where\theta =\left[\begin{array}{c} w_{1}\\ \vdots \\ w_{e}\\ b_{1}\\ \vdots \\ b_{e} \end{array}\right]$$

(33) $$\theta^{i}=\theta^{i-1}-\eta \nabla L\left(\theta^{i-1}\right)where\nabla L\left(\theta^{i-1}\right)=\left[\begin{array}{c} \frac{\partial L\left(\theta^{i-1}\right)}{\partial w_{1}}\\ \vdots \\ \frac{\partial L\left(\theta^{i-1}\right)}{\partial w_{e}}\\ \frac{\partial L\left(\theta^{i-1}\right)}{\partial b_{1}}\\ \vdots \\ \frac{\partial L\left(\theta^{i-1}\right)}{\partial b_{e}} \end{array}\right]$$

where *i* denotes the iteration number, and *η* is the learning rate parameter. By setting the optimizer as Adam [143], we trained our DTI model with learning rate *η* = 0.001, epoch = 100, and batch size = 100 samples. To counter overfitting, an early stopping strategy was employed to monitor the validation error at each epoch and stop the model training once errors started to increase. Moreover, we set the dropout layer after each hidden layer in the DNN-based DTI model architecture and set 0.5 for the dropout rate. All the DNN-based DTI model construction and training processes were conducted by using Tensorflow and Keras package under the Python 3.7 environment on a computer with an Intel i7-8550U 3.4 GHz processor and 32 GB memory.

The common method for evaluating the quality of a binary classifier is to plot the receiver operating characteristic (ROC) curve and measure the area under the curve (AUC-ROC) [205]. The ROC curve can be created by plotting the true positive rate (TPR) against the false positive rate (FPR) at every probability threshold. In general, the AUC-ROC score of a perfect classifier will equal 1, whereas the AUC-ROC score of a purely random classifier will equal 0.5. Therefore, measuring the AUC-ROC score can be used to compare the performance of different classifiers. The formulas of the AUC-ROC curve are shown in the following Equations

(34) $$TruePositiveRate(TPR)=Sensitivity=Recall=\frac{TruePositive(TP)}{TruePositive(TP)+FalseNegative(FN)}$$

(35) $$TrueNegativeRate(TNR)=Specificity=\frac{TrueNegative(TN)}{TrueNegative(TN)+FalsePositive(FP)}$$

(36) $$FalsePositiveRate(FPR)=1-Specificity=\frac{FalsePositive(FP)}{FalsePositive(FP)+TrueNegative(TN)}$$

where true positive (TP) is the outcome model that correctly predicts value in the positive class; true negative (TN) is the outcome model that correctly predicts value in the negative class; false positive (FP) is the outcome model that incorrectly predicts actual value in the positive class; false negative (FN) is the outcome model that incorrectly predicts actual value in the negative class.

## Figures and Tables

**Table 1:** Sample groups and statistics of nodes in integrated datasets collected from RNA-Seq datasets of Gene Expression Omnibus (GEO) database (accession nos. GSE156063 and GSE163151).

	**Datasets**	**GSE163151**	**GSE156063**	**Integrated**	**Group Definition**
**Sample**					
COVID-19-associated ARDS		138	93	231	ARDS patients caused by SARS-CoV-2 infection
Non-viral ARDS		82	100	182	ARDS patients not caused by viral infection (including SARS-CoV-2)
	**Datasets**	**GSE163151**	**GSE156063**	**Integrated**	**Node Description**
**Nodes**					
*Protein*		17055	12929	18225	Nodes with unknown functions (excluding *Rcp, TF, miRNA, LncRNA,* and *Virus*) are assumed to express protein.
*Rcp*		2484	1700	2500	Receptor
*TF*		1502	1216	1519	Transcription factor
*RcpTF*		105	89	105	Nodes with both *Rcp* and *TF* function
*miRNA*		1378	0	1378	miRNA
*LncRNA*		2781	35	2784	LncRNA
*Virus*		0	13	13	SARS-CoV-2 nodes (please refer to Table S1 for detail)
Total		24309	15982	26524	

NOTE: Nodes are proteins/genes that have at least 1 interaction with others in the network. For the convenience of analysis, nodes are classified into 7 classes (*Protein, Rcp, TF, RcpTF, miRNA, LncRNA, Virus*) in this study.

**Table 2:** Comparison of numbers of nodes in candidate HPI-GWGEN, real HPI-GWGEN of COVID-19-associated ARDS, and real HPI-GWGEN of non-viral ARDS after system identification.

Nodes	Candidate HPI-GWGEN		Real HPI-GWGEN (Non-Viral ARDS)		Real HPI-GWGEN (COVID-19-Associated ARDS)	
	HPI-PPI	HPI-GRN	HPI-PPI	HPI-GRN	HPI-PPI	HPI-GRN
*Proteins*	18,225	18,225	15,287	11,055	18,111	12,027
*Rcp*	2500	2500	2228	1859	2469	1959
*TF*	1519	1519	1374	1120	1511	1191
*RcpTF*	105	105	96	93	103	95
*miRNA*	0	1378	0	809	0	799
*LncRNA*	0	2784	0	1934	0	2116
*Virus*	11	13	0	0	11	13
Total	22,360	26,524	18,985	16,870	22,205	18,200

NOTE: Nodes are proteins/genes that have at least 1 interaction with others in the network. For the convenience of analysis, nodes are classified into 7 classes in this study.

**Table 3:** Comparison of numbers of edges in candidate HPI-GWGEN, real HPI-GWGEN of COVID-19-associated ARDS, and real HPI-GWGEN of non-viral ARDS after system identification.

Edges	Candidate HPI-GWGEN		Real HPI-GWGEN (Non-Viral ARDS)		Real HPI-GWGEN (COVID-19-Associated ARDS)	
	HPI-PPI	HPI-GRN	HPI-PPI	HPI-GRN	HPI-PPI	HPI-GRN
*Proteins* _↔_ *Proteins*	3,013,811	222,665	1,400,482	128,900	1,445,193	124,144
*Proteins* _↔_ *Rcp*	828,208	48,644	360,024	26,382	375,835	25,551
*Proteins* _↔_ *TF*	455,807	20,823	219,681	12,044	234,620	11,616
*Proteins* _↔_ *RcpTF*	21,098	2763	12,461	1642	12,905	1673
*Proteins* _↔_ *miRNA*	0	34,039	0	9094	0	8458
*Proteins* _↔_ *LncRNA*	0	60,640	0	28,705	0	27,298
*Proteins* _↔_ *Virus*	200,475	236,925	0	0	117,139	1999
*Rcp* _↔_ *Rcp*	56,203	1088	22,761	520	24,157	491
*Rcp* _↔_ *TF*	62,965	537	28,875	267	31,100	247
*Rcp* _↔_ *RcpTF*	2977	73	1679	38	1766	35
*Rcp* _↔_ *miRNA*	0	2585	0	404	0	365
*Rcp* _↔_ *LncRNA*	0	3559	0	1256	0	1265
*Rcp* _↔_ *Virus*	27,500	32,500	0	0	14,958	306
*TF* _↔_ *TF*	15,427	18	7983	11	8893	12
*TF* _↔_ *RcpTF*	1677	9	1093	5	1229	7
*TF* _↔_ *miRNA*	0	1218	0	164	0	197
*TF* _↔_ *LncRNA*	0	1476	0	546	0	629
*TF* _↔_ *Virus*	16,709	19,747	0	0	10,003	203
*RcpTF* _↔_ *RcpTF*	9	1	6	1	6	1
*RcpTF* _↔_ *miRNA*	0	132	0	14	0	20
*RcpTF* _↔_ *LncRNA*	0	145	0	50	0	58
*RcpTF* _↔_ *Virus*	1155	1365	0	0	750	14
*miRNA* _↔_ *miRNA*	0	1039	0	36	0	36
*miRNA*_↔_ *LncRNA*	0	3340	0	803	0	586
*miRNA* _↔_ *Virus*	0	17,914	0	0	0	28
*LncRNA* _↔_ *LncRNA*	0	2633	0	1139	0	1109
*LncRNA* _↔_ *Virus*	0	36,192	0	0	0	383
*Virus* _↔_ *Virus*	66	91	0	0	4	0
Total (PPI/GRN)	4,704,087	752,161	2,055,045	212,021	2,278,558	206,762
Total (PPI+GRN)	5,456,248		2,267,066		2,485,320	

NOTE: Edges are defined as interactions between 2 nodes and expressed with” node1 ↔ node2”, where “node1” and “node2” are gene/protein names from one of the 7 classes we defined.

**Table 4:** Potential small molecule compounds selected for each identified biomarker based on the drug design specifications.

**TNF (+)**								
Drug	Regulation Ability (L1000)	Sensitivity (PRISM)	Toxicity (LC_50_, mol/kg)	Clearance (CL, mL/min/kg)	Drug-Likeness			
					Lipinski Rule	Pfizer Rule	GSK Rule	Golden Triangle
Nicorandil	−0.077	0.039	3.316	8.271	Accepted	Accepted	Accepted	Accepted
Eugenol	−0.321	−0.067	3.926	14.042	Accepted	Accepted	Accepted	Rejected
Omeprazole	−0.132	−0.050	3.570	5.938	Accepted	Accepted	Accepted	Accepted
Niclosamide	−0.264	0.213	5.631	1.681	Accepted	Accepted	Rejected	Accepted
Nimodipine	−0.228	−0.349	4.584	12.024	Accepted	Accepted	Rejected	Accepted
**NFkB (+)**								
Drug	Regulation ability (L1000)	Sensitivity (PRISM)	Toxicity (LC_50_, mol/kg)	Clearance (CL, mL/min/kg)	Drug-likeness			
					Lipinski Rule	Pfizer Rule	GSK Rule	Golden Triangle
Nicorandil	−0.330	0.039	3.316	8.271	Accepted	Accepted	Accepted	Accepted
Isoliquiritigenin	−0.304	−0.139	6.091	14.805	Accepted	Accepted	Accepted	Accepted
Omeprazole	−0.180	−0.050	3.570	5.938	Accepted	Accepted	Accepted	Accepted
Calcipotriol	−0.273	−0.309	5.777	1.110	Accepted	Accepted	Rejected	Accepted
Sitagliptin	−0.220	−0.102	2.704	5.894	Accepted	Accepted	Rejected	Accepted
**HIF1A (+)**								
Drug	Regulation ability (L1000)	Sensitivity (PRISM)	Toxicity (LC_50_, mol/kg)	Clearance (CL, mL/min/kg)	Drug-likeness			
					Lipinski Rule	Pfizer Rule	GSK Rule	Golden Triangle
Nicorandil	−0.876	0.039	3.316	8.271	Accepted	Accepted	Accepted	Accepted
Isoliquiritigenin	−0.548	−0.139	6.091	14.805	Accepted	Accepted	Accepted	Accepted
Naftopidil	−0.377	0.407	4.735	11.276	Accepted	Rejected	Rejected	Accepted
Valsartan	−0.253	0.132	3.149	0.314	Accepted	Accepted	Rejected	Accepted
Alvocidib	−0.173	−4.405	5.608	5.810	Accepted	Accepted	Rejected	Accepted
**HSPA5 (+)**								
Drug	Regulation ability (L1000)	Sensitivity (PRISM)	Toxicity (LC_50_, mol/kg)	Clearance (CL, mL/min/kg)	Drug-likeness			
					Lipinski Rule	Pfizer Rule	GSK Rule	Golden Triangle
Isoliquiritigenin	−0.493	−0.139	6.091	14.805	Accepted	Accepted	Accepted	Accepted
Metformin	−0.496	0.371	2.039	3.504	Accepted	Accepted	Accepted	Rejected
Phenformin	−0.317	−0.415	2.622	8.273	Accepted	Accepted	Accepted	Accepted
Losartan	−0.289	0.084	6.961	10.673	Accepted	Accepted	Rejected	Accepted
Purvalanol-b	−0.159	0.178	3.465	6.333	Accepted	Accepted	Rejected	Accepted
**FTO (+)**								
Drug	Regulation ability (L1000)	Sensitivity (PRISM)	Toxicity (LC_50_, mol/kg)	Clearance (CL, mL/min/kg)	Drug-likeness			
					Lipinski Rule	Pfizer Rule	GSK Rule	Golden Triangle
Mefenamic-acid	−0.980	−0.145	4.109	1.419	Accepted	Rejected	Rejected	Accepted
Omeprazole	−0.361	−0.050	3.570	5.938	Accepted	Accepted	Accepted	Accepted
Tozasertib	−0.284	−0.364	3.773	2.528	Accepted	Accepted	Rejected	Accepted
Dicloxacillin	−0.194	0.006	4.353	1.829	Accepted	Accepted	Rejected	Accepted
Lovastatin	−0.103	0.796	3.792	17.025	Accepted	Accepted	Rejected	Accepted
**BECN1 (+)**								
Drug	Regulation ability (L1000)	Sensitivity (PRISM)	Toxicity (LC_50_, mol/kg)	Clearance (CL, mL/min/kg)	Drug-likeness			
					Lipinski Rule	Pfizer Rule	GSK Rule	Golden Triangle
Eugenol	−0.283	−0.067	3.926	14.042	Accepted	Accepted	Accepted	Rejected
Omeprazole	−0.136	−0.050	3.570	5.938	Accepted	Accepted	Accepted	Accepted
Tacedinaline	−0.135	−0.681	3.772	1.313	Accepted	Accepted	Accepted	Accepted
Pevonedistat	−0.109	−1.667	6.855	8.914	Accepted	Accepted	Rejected	Accepted
Danusertib	−0.091	−2.448	2.357	3.461	Accepted	Accepted	Rejected	Accepted
**FOXA1 (+)**								
Drug	Regulation ability (L1000)	Sensitivity (PRISM)	Toxicity (LC_50_, mol/kg)	Clearance (CL, mL/min/kg)	Drug-likeness			
					Lipinski Rule	Pfizer Rule	GSK Rule	Golden Triangle
Olaparib	−1.109	0.012	2.976	3.522	Accepted	Accepted	Rejected	Accepted
Bortezomib	−0.018	−2.783	2.474	2.742	Accepted	Accepted	Accepted	Accepted
Carvedilol	−0.015	0.389	5.014	8.419	Accepted	Accepted	Rejected	Accepted
Desoxypeganine	−0.014	−0.081	2.952	6.957	Accepted	Accepted	Accepted	Rejected
Valsartan	−0.004	0.132	3.149	0.314	Accepted	Accepted	Rejected	Accepted
Ipsapirone	−0.003	−0.235	2.823	2.248	Accepted	Accepted	Rejected	Accepted

(+), abnormal overexpression; (−), abnormal low expression.

**Table 5:** Details information of drug-likeness filters.

	Description	Note
Lipinski rules	MW ≤ 500, logP ≤ 5, H-bound acceptors ≤ 10, H-bound receptors ≤ 5	If more than 2 properties are out of range, poor absorption or permeability may occur.
Pfizer rules	logP > 3, TPSA < 75	Compounds satisfying the Pfizer rules imply that they are more likely to be toxic.
GSK rule	MW ≤ 400, logP ≤ 4	In general, compounds satisfying the Golden Triangle and GSK rule usually have a favorable ADMET (absorption, distribution, metabolism, excretion, toxicity) profile
Golden Triangle	200 ≤ MW ≤ 50, −2 ≤ logD ≤ 5	

Abbreviations: MW, molecular weight (unit, Da); logP, distribution coefficient P; logD, n-octanol/water distribution coefficients; TPSA, topological polar surface area.

**Table 6:** Selected drugs and their corresponding drug targets in the multiple-molecule drug therapy for COVID-19-associated ARDS.

	Targets	TNF	NFkB	HIF1A		GRP78	FTO	BECN1
Drugs								
Nicorandil		⬤	⬤	⬤				
Isoliquiritigenin			⬤	⬤		⬤		
Eugenol		⬤						⬤
Omeprazole		⬤	⬤				⬤	⬤
Chemical structures of multiple-molecule drug								
Nicorandil					Isoliquiritigenin			
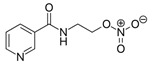					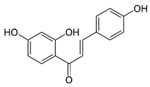			
Eugenol					Omeprazole			
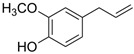					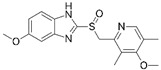			

⬤: The proposed small molecules to target the biomarkers (drug targets).

**Table 7:** Selected drugs and their corresponding drug targets in multiple-molecule drug therapy for non-viral ARDS.

	Targets	TNF	NFkB		HIF1A	FOXA1
Drugs						
Nicorandil		⬤	⬤		⬤	
Bortezomib			⬤			⬤
Olaparib						⬤
Chemical structures of multiple-molecule drug						
Nicorandil				Bortezomib		
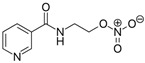				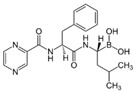		
Olaparib						
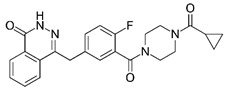						

⬤: The proposed small molecules to target the biomarkers (drug targets).

## Data Availability

The gene raw counts datasets of human genes are integrated from GSE156063 (https://www.ncbi.nlm.nih.gov/geo/query/acc.cgi?acc=GSE156063) (accessed on 28 November 2021) and GSE163151 (https://www.ncbi.nlm.nih.gov/geo/query/acc.cgi?acc=GSE163151) (accessed on 28 November 2021). RNA-seq raw data files of GSE156063 (removed human reads) can be downloaded from PRJNA633853 (https://www.ebi.ac.uk/ena/browser/view/PRJNA633853?show=reads) (accessed on 28 November 2021). Drug regulation ability data is from Phase I L1000 Level 5 datasets (https://www.ncbi.nlm.nih.gov/geo/query/acc.cgi?acc=GSE92742)(accessed on 28 November 2021). Drug sensitivity datasets are from DepMapPRISM primary screen datasets (https://depmap.org/repurposing/)(accessed on 28 November 2021).

## Supplementary Materials

The following supporting information can be downloaded at: https://www.mdpi.com/article/10.3390/ijms23073649/s1.
