# Supplementary material for: Repurposing Multiple-Molecule Drugs for COVID-19-Associated Acute Respiratory Distress Syndrome and Non-Viral Acute Respiratory Distress Syndrome via a Systems Biology Approach and a DNN-DTI Model Based on Five Drug Design Specifications
Source: Int J Mol Sci. 2022 Mar 26;23(7):3649. doi: 10.3390/ijms23073649 (PMC8998971; doi:10.3390/ijms23073649)
Supplement: Supplementary file 1 [file ijms-23-03649-s001.zip › ijms-1633967-supplementary.pdf]

Supplementary materials

1.1 Systematic Model Construction for miRNAs and lncRNAs in Candidate HPI-GWGEN of COVID-19-associated ARDS and Non-Viral-ARDS Patient

The lth host lncRNA gene in the GRN interaction model can be described by the following equation:

$$l_l^H[n] = \sum_{\tau=1}^{T_j} T_{l\tau}^H t_\tau^H[n] - \sum_{\mu=1}^{M_l} M_{l\mu}^H l_l^H[n] m_\mu^H[n] + \sum_{\lambda=1,\lambda\neq l}^{L_l} L_{l\lambda}^H l_\lambda^H[n] + \beta_{l,GRN}^H + \varepsilon_{l,GRN}^H[n] \tag{1}$$
$$-M_{l\mu}^H \le 0 \qquad for\ l = 1 \sim L\ , n = 1 \sim N$$

where $l_l^H[n], t_\tau^H[n], l_\lambda^H[n], m_\mu^H[n]$ indicate the expression level of the lth host lncRNA gene, the τth host TF, the lth host lncRNA gene, and the rth host miRNA gene in the nth sample, respectively; $T_{l\tau}^H, L_{l\lambda}^H, M_{l\mu}^H$ indicate the regulation ability of the τth host TF, the λth host lncRNA gene and the μth host miRNA gene on the lth host lncRNA gene, respectively; $\beta_{l,GRN}^H$ indicates the basal level of the lth host lncRNA gene in the nth sample; $\varepsilon_{l,GRN}^H[n]$ indicates the stochastic noise of the lth host lncRNA gene in the nth sample; T_l, L_l, M_l indicate the total number of host TF, host lncRNA gene and host miRNA gene interacting with the lth host lncRNA gene, respectively; L indicates the total number of the lth host lncRNA gene in candidate GRN; N denotes sample number in candidate GRN, either in COVID-19-associated ARDS or Non-Viral-ARDS group.

The μth host miRNA gene in the GRN interaction model can be described by the following equation:

$$m_{\mu}^{H}[n]=\sum_{\tau=1}^{T_{\mu}}T_{\mu\tau}^{H}t_{\tau}^{H}[n]-\sum_{x=1,x\neq\mu}^{M_{\mu}}M_{\mu x}^{H}m_{\mu}^{H}[n]m_{x}^{H}[n]+\sum_{\lambda=1}^{L_{\mu}}L_{\mu\lambda}^{H}l_{\lambda}^{H}[n]+\beta_{\mu,GRN}^{H}+\varepsilon_{\mu,GRN}^{H}[n] \quad (2)$$
$$-M_{\mu x}^{H}\leq 0 \qquad for\ \mu=1\sim M\ ,n=1\sim N$$

where $m_{\mu}^{H}[n],t_{\tau}^{H}[n],l_{\lambda}^{H}[n],m_{x}^{H}[n]$ indicate the expression level of the μth host miRNA gene, the τth host TF, the λth host lncRNA gene, and the xth host miRNA gene in the nth sample, respectively; $T_{\mu\tau}^{H},L_{\mu\lambda}^{H},M_{\mu x}^{H}$ indicate the regulation ability of the τth host TF, the λth host lncRNA gene and the xth host miRNA gene on the μth host miRNA gene, respectively; $\beta_{\mu,GRN}^{H}$ indicate the basal level of the μth host miRNA gene in the nth sample; $\varepsilon_{\mu,GRN}^{H}[n]$ indicates the stochastic noise of the μth host miRNA gene in the nth sample; T_{μ},L_{μ},M_{μ} indicate the total number of host TF, host lncRNA gene and host miRNA gene interacting with the μth host miRNA gene, respectively; M indicates the total number of the μth host miRNA gene in candidate GRN; N denotes sample number in candidate GRN, either in COVID-19-associated ARDS or Non-Viral-ARDS group.

1.2 Systems identification and model order selection for obtaining real GWGENs of COVID-19-associated ARDS and Non-Viral-ARDS

Equations (1) ~ (2) can be expressed as the following regression form, respectively:

$$l_{l}^{H}[n]=\begin{bmatrix}t_{1}^{H}[n] & L & t_{T_l}^{H}[n] & l_{1}^{H}[n]m_{1}^{H}[n] & L & l_{l}^{H}[n]m_{M_l}^{H}[n] & l_{1}^{H}[n] & L & l_{L_l}^{H}[n] & 1\end{bmatrix}\times\begin{bmatrix}T_{l1}^{H}\\ M\\ T_{lT_l}^{H}\\ -M_{l1}^{H}\\ M\\ -M_{lM_l}^{H}\\ L_{l1}^{H}\\ M\\ L_{lL_l}^{H}\\ \beta_{l,GRN}^{H}\end{bmatrix}+\varepsilon_{l,GRN}^{H}[n] \quad (3)$$
$$=\varphi_{l}^{HL}[n]\theta_{l}^{HL}+\varepsilon_{l,GRN}^{H}[n]\quad ,\ for\ l=1\sim L\ ,n=1\sim N$$

$$m_{\mu}^{H}[n] = \left[t_{1}^{H}[n] \;\; \cdots \;\; t_{T_{\mu}}^{H}[n] \;\; m_{\mu}^{H}[n] m_{1}^{H}[n] \;\; \cdots \;\; m_{\mu}^{H}[n] m_{M_{\mu}}^{H}[n] \;\; l_{1}^{H}[n] \;\; \cdots \;\; l_{L_{\mu}}^{H}[n] \;\; 1 \right] \times \begin{bmatrix} T_{\mu 1}^{H} \\ \vdots \\ T_{\mu T_{\mu}}^{H} \\ -M_{\mu 1}^{H} \\ \vdots \\ -M_{\mu M_{\mu}}^{H} \\ L_{\mu 1}^{H} \\ \vdots \\ L_{\mu L_{\mu}}^{H} \\ \beta_{\mu,GRN}^{H} \end{bmatrix} + \varepsilon_{\mu,GRN}^{H}[n] \tag{4}$$

$$= \varphi_{\mu}^{HM}[n] \theta_{\mu}^{HM} + \varepsilon_{\mu,GRN}^{H}[n] \quad , \; for \; \mu = 1 \sim M \; , n = 1 \sim N$$

where the superscript *H, P, HL,* and *HM* denote abbreviation of the host, pathogen, host lncRNA gene, host miRNA gene, and pathogen gene, respectively; $\boldsymbol{\varphi}_{l}^{HL}[n], \boldsymbol{\varphi}_{\mu}^{HM}[n]$ denote the regression vector which can be obtained from the corresponding expression data we integrated; $\boldsymbol{\theta}_{l}^{HL}, \boldsymbol{\theta}_{\mu}^{HM}$ are corresponding unknown parameter vectors of the lth host lncRNA gene and the μth host miRNA gene, respectively.

Equation (3) ~ (4) can be further augmented for N samples as follows

$$\begin{bmatrix} l_{l}^{H}[1] \\ l_{l}^{H}[2] \\ \vdots \\ l_{l}^{H}[N] \end{bmatrix} = \begin{bmatrix} \varphi_{l}^{HL}[1] \\ \varphi_{l}^{HL}[2] \\ \vdots \\ \varphi_{l}^{HL}[N] \end{bmatrix} \theta_{l}^{HL} + \begin{bmatrix} \varepsilon_{l,GRN}^{H}[1] \\ \varepsilon_{l,GRN}^{H}[2] \\ \vdots \\ \varepsilon_{l,GRN}^{H}[N] \end{bmatrix} \quad for \; l = 1 \sim L \tag{5}$$

$$\begin{bmatrix} m_{\mu}^{H}[1] \\ m_{\mu}^{H}[2] \\ \vdots \\ m_{\mu}^{H}[N] \end{bmatrix} = \begin{bmatrix} \varphi_{\mu}^{HM}[1] \\ \varphi_{\mu}^{HM}[2] \\ \vdots \\ \varphi_{\mu}^{HM}[N] \end{bmatrix} \theta_{\mu}^{HM} + \begin{bmatrix} \varepsilon_{\mu,GRN}^{H}[1] \\ \varepsilon_{\mu,GRN}^{H}[2] \\ \vdots \\ \varepsilon_{\mu,GRN}^{H}[N] \end{bmatrix} \quad for \; \mu = 1 \sim M \tag{6}$$

Equations (5) ~ (6) above can be simply represented as follows

$$\boldsymbol{L}_{l}^{H} = \boldsymbol{\Phi}_{l}^{HL} \theta_{l}^{HL} + \boldsymbol{\Omega}_{l}^{HL} \quad , for \; l = 1 \sim L \tag{7}$$

$$\boldsymbol{M}_{\mu}^{H} = \boldsymbol{\Phi}_{\mu}^{HM} \theta_{\mu}^{HM} + \boldsymbol{\Omega}_{\mu}^{HM} \quad , for \; \mu = 1 \sim M \tag{8}$$

For each parameter vector $\theta_{l}^{HL}, \theta_{\mu}^{HM}$ in equations (7) ~ (8) , we can individually

estimate by solving the constrained least-square problem as follows:

$$\begin{aligned} &\hat{\theta}_l^{HL} = \underset{\theta_l^{HL}}{argmin}\,\frac{1}{2}\left\| \boldsymbol{\Phi}_l^{HL}\theta_l^{HL} - \boldsymbol{L}_l^{H} \right\|_2^2 \quad , subject\ to\ \boldsymbol{A}_l^{HL}\hat{\theta}_l^{HL} \le \boldsymbol{B}_l^{HL} \\ &where\ \boldsymbol{A}_l^{HL} = \left[\boldsymbol{O}_{M_l \times T_l} \;\vdots\; \boldsymbol{I}_{M_l \times M_l} \;\vdots\; \boldsymbol{O}_{M_l \times L_l} \;\vdots\; \boldsymbol{O}_{M_l \times 1} \right] \quad , \boldsymbol{B}_l^{HL} = \left[\boldsymbol{O}_{M_l \times 1} \right] \end{aligned} \tag{9}$$

$$\begin{aligned} &\hat{\theta}_\mu^{HM} = \underset{\theta_\mu^{HM}}{argmin}\,\frac{1}{2}\left\| \boldsymbol{\Phi}_\mu^{HM}\theta_\mu^{HM} - \boldsymbol{M}_\mu^{H} \right\|_2^2 \quad , subject\ to\ \boldsymbol{A}_\mu^{HM}\hat{\theta}_\mu^{HM} \le \boldsymbol{B}_\mu^{HM} \\ &where\ \boldsymbol{A}_\mu^{HM} = \left[\boldsymbol{O}_{M_\mu \times T_\mu} \;\vdots\; \boldsymbol{I}_{M_\mu \times M_\mu} \;\vdots\; \boldsymbol{O}_{M_\mu \times L_\mu} \;\vdots\; \boldsymbol{O}_{M_\mu \times 1} \right] \quad , \boldsymbol{B}_\mu^{HM} = \left[\boldsymbol{O}_{M_\mu \times 1} \right] \end{aligned} \tag{10}$$

where $\boldsymbol{O}$ and $\boldsymbol{I}$ denote zero matrix and identity matrix, respectively.

It is noted that in the parameter fitting process for the regression model of each protein/gene, what candidate HPI-GWGEN provided is all possible binding molecules and our model will need further parameter trimming process. However, such a model parameter identification process in equations (9) ~ (10) will often result in overfitting conditions when a finite sample of the dataset at hand. Therefore, the sys-tems order detection method, AIC, was employed to detect the system order (i.e., the number of interactions of each protein with other proteins or the number of regulator TFs on each gene by the fact that system order can minimize the corresponding AIC). For each model in HPI-GWGEN, the AIC values of the lth host lncRNA gene in equation (1) and the μth host miRNA gene in equation (2) are defined as follows:

$$AIC_l^{HL}(\hat{\theta}_l^{HL}, \boldsymbol{\Phi}_l^{HL}, \boldsymbol{L}_l^{H}) = log\left(\frac{\left\| \boldsymbol{\Phi}_l^{HL}\hat{\theta}_l^{HL} - \boldsymbol{L}_l^{H} \right\|_2^2}{N}\right) + \frac{2dim(\hat{\theta}_l^{HL})}{N} \quad , for\ l = 1 \sim L \tag{11}$$

$$AIC_\mu^{HM}(\hat{\theta}_\mu^{HM}, \boldsymbol{\Phi}_\mu^{HM}, \boldsymbol{M}_\mu^{H}) = log\left(\frac{\left\| \boldsymbol{\Phi}_\mu^{HM}\hat{\theta}_\mu^{HM} - \boldsymbol{M}_\mu^{H} \right\|_2^2}{N}\right) + \frac{2dim(\hat{\theta}_\mu^{HM})}{N} \quad , for\ \mu = 1 \sim M \tag{12}$$

where $dim(\tilde{\theta}_l^{HL})$ and $dim(\tilde{\theta}_\mu^{HM})$ denote parameter vector dimension of each model, respectively. In general, increasing parameter number (system order) will result in good model fit, such that log residual error in the first term of AIC will decrease and the second term of AIC will increase, and vice versa. Therefore, there should be exactly parameter numbers corresponding with the optimal parameter vector to achieve the minimum AIC among all possible binding combinations for each protein/gene. Considering practical computational efficiency for implementation, for each protein/gene, forward and backward stepwise algorithms are both adopted to find the minimum AIC in equation (11) ~ (12), with the corresponding parameter numbers to achieve the minimum AIC in equation (9) ~ (10) with the help of *lsqlin* function in 2021 MATLAB optimization toolbox. Therefore, we trimmed the insignificant parameters in candidate HPI-GWGEN out of system order detected by AIC to obtain real HPI-GWGEN of COVID-19-associated ARDS and Non-Viral-ARDS.

Tables

Table S1. Details of nodes included in *Virus* class used for SARS-CoV-2 in HPI-PPI and HPI-GRN.

Node / Network	*Virus*	Total
HPI-PPI	E, M, N, ORF10, ORF1ab, ORF3a, ORF6, ORF7a, ORF7b, ORF8, S	11
HPI-GEN	3UTR, 5UTR, E, M, N, ORF10, ORF1ab, ORF3a, ORF6, ORF7a, ORF7b, ORF8, S	13

Note: **5UTR** 5′-untranslated region; **3UTR** 5′-untranslated region; **ORF1ab** in HPI-PPI is polyprotein which can be further cleaved to yield 16 nonstructural proteins.

Table S2. KEGG pathway enrichment analysis of COVID-19-associated ARDS core HPI-GWGEN using DAVID website.

Term	Gene Numbers	*p*-value
Olfactory transduction	101	4.04E-11
Autoimmune thyroid disease	19	6.86E-05
Neuroactive ligand-receptor interaction	57	6.38E-04
Graft-versus-host disease	13	6.70E-04
Cell adhesion molecules (CAMs)	33	1.51E-03
Allograft rejection	13	2.11E-03
Type I diabetes mellitus	14	2.24E-03
Hypertrophic cardiomyopathy (HCM)	19	1.19E-02
Cytokine-cytokine receptor interaction	45	1.77E-02
Dilated cardiomyopathy	19	2.49E-02
Endometrial cancer	13	3.62E-02

Note: KEGG PATHWAY is a collection of molecular pathway diagrams, which are categorized by the annotation terms to represent the current knowledges about the metabolism or cellular function of each species. This table summarizes the functional annotation chart and is obtained by submitting the genes list of 4000 nodes to the DAVID website. Each annotation term summarized total gene counts involved in the corresponding KEGG pathway among the 4000 genes we upload.

Table S3. The KEGG pathway enrichment analysis of Non-Viral-ARDS core HPI-GWGEN using DAVID website.

Term	Gene Numbers	*p*-value
Olfactory transduction	106	4.30E-11
Amoebiasis	28	1.46E-03
Mineral absorption	15	2.28E-03
Protein digestion and absorption	23	4.95E-03
Bile secretion	19	6.58E-03
Platelet activation	30	8.01E-03

Leukocyte transendothelial migration	27	9.78E-03
Pertussis	19	1.59E-02
ECM-receptor interaction	21	1.83E-02
RIG-I-like receptor signaling pathway	17	3.42E-02

Note: KEGG PATHWAY is a collection of molecular pathway diagrams, which are categorized by the annotation terms to represent the current knowledges about the metabolism or cellular function of each species. This table summarizes the functional annotation chart and is obtained by submitting the genes list of 4000 nodes to the DAVID website. Each annotation term summarized total gene counts involved in the corresponding KEGG pathway among the 4000 genes we upload.

Table S4. Model performance of DNN-DTI model (5-fold cross-validation, epoch=52).

	Validation loss	Validation Accuracy	Testing loss	Testing Accuracy
1	0.185257	0.932791	0.230130	0.932164
2	0.205787	0.930211	0.192034	0.930566
3	0.197644	0.927939	0.201237	0.929030
4	0.214519	0.929585	0.204637	0.932890
5	0.201079	0.934167	0.213735	0.931209
Average	0.200857	0.930938	0.208355	0.931172
Standard Deviation	0.009640	0.002245	0.012915	0.001334

Figures

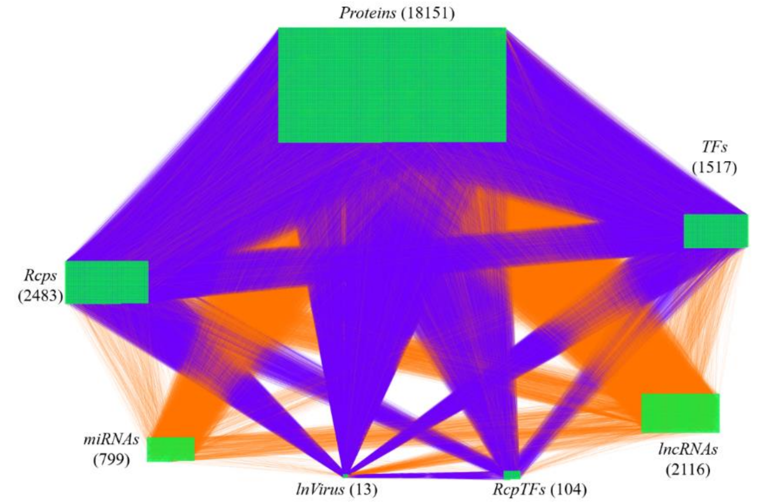

Figure S1. The host-pathogen interspecies real genome-wide genetic and epigenetic network (HPI-GWGEN) of COVID-19-associated ARDS. Purple lines indicate the protein-protein interactions; Orange lines denote the gene regulations. The numbers of *Proteins, Rcps, TFs, RcpTFs, miRNA, LncRNA, Virus* are 18151, 2483, 104, 799, 2116, and 13, respectively.

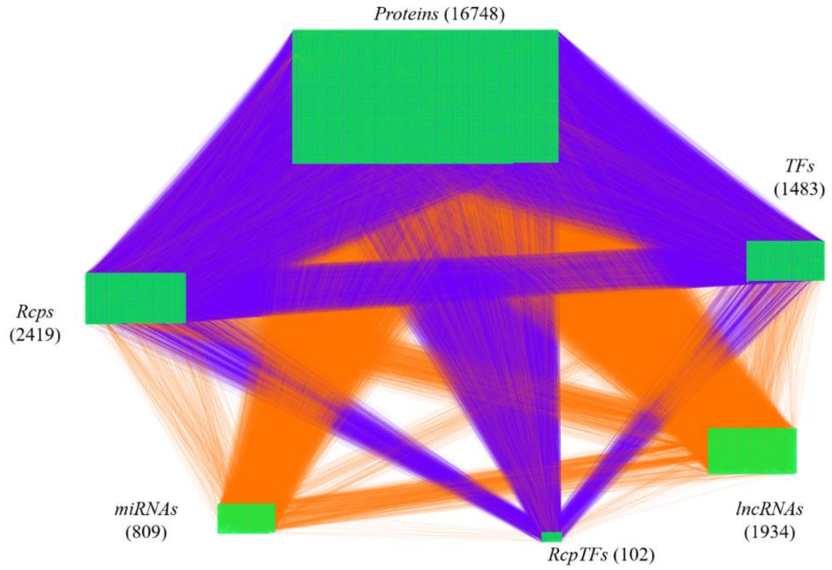

Figure S2. The host-pathogen interspecies real genome-wide genetic and epigenetic network (HPI-GWGEN) of Non-Viral-ARDS. Purple lines indicate the protein-protein interactions; Orange lines denote the gene regulations. The numbers of *Proteins, recptors, TFs, RcpTFs, miRNA, and LncRNA* are 16748, 2419, 1483, 102, 8099, and 1934, respectively

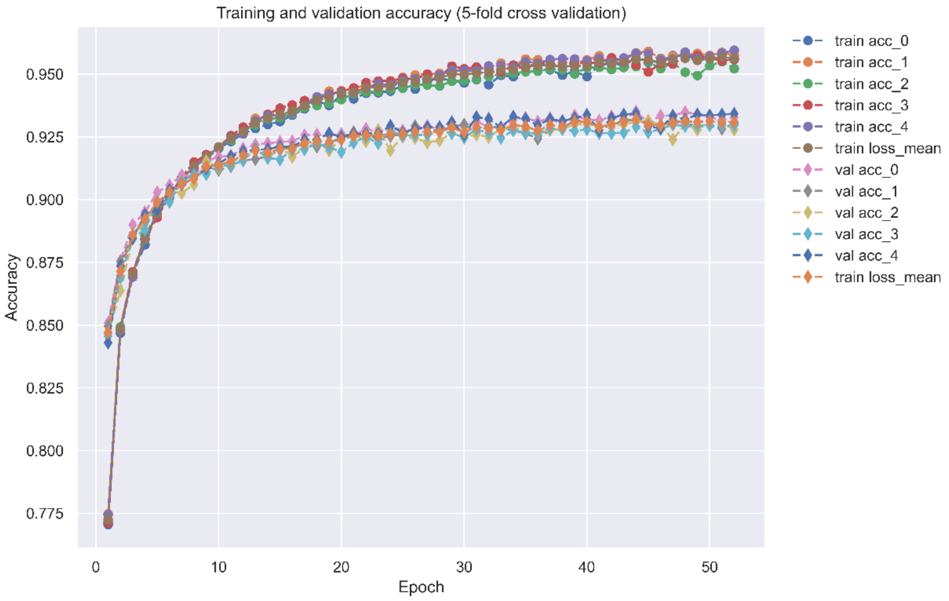

Figure S3. Accuracy learning curves of deep neuron network drug-target interaction model (DNN-DTI). The early Stopping strategy is applied to automatically stop the learning process at the epoch of 52.

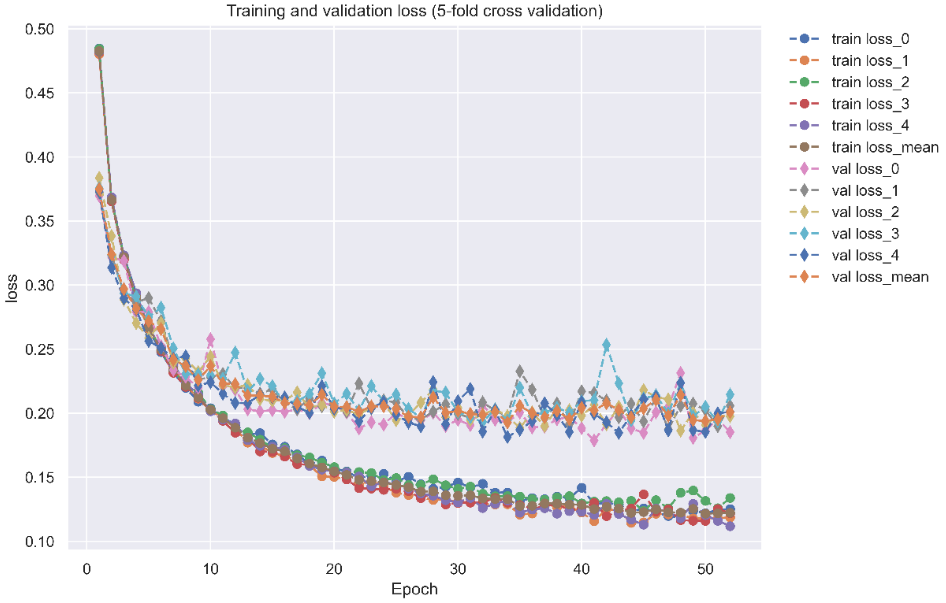

Figure S4. Loss learning curves of deep neuron network drug-target interaction model (DNN-DTI). The early Stopping strategy is applied to automatically stop the learning process at the epoch of 52.

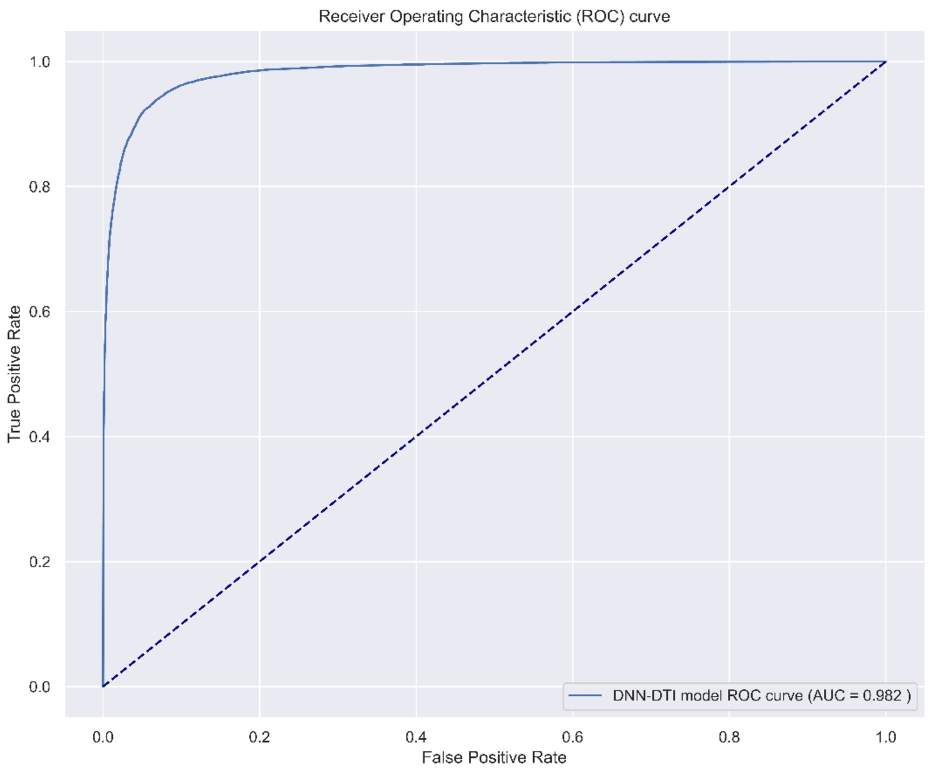

Figure S5. The Receiver Operating Characteristic (ROC) curve and the Area Under the Curve of ROC (AUC-ROC) score of the deep neuron network drug-target interaction model (DNN-DTI). A purple diagonal dot line indicates the worst situation when AUC is approximately 0.50, where the model has no discrimination capacity to distinguish between positive and negative classes. A classifier that predicts at random will appear as the diagonal line. The dotted line represents the ROC curve of a purely random classifier.
